# Investigation of Endoglin Wild-Type and Missense Mutant Protein Heterodimerisation Using Fluorescence Microscopy Based IF, BiFC and FRET Analyses

**DOI:** 10.1371/journal.pone.0102998

**Published:** 2014-07-31

**Authors:** Tassilo Förg, Mathias Hafner, Andreas Lux

**Affiliations:** 1 Institute for Molecular and Cell Biology, University of Applied Sciences, Mannheim, Germany; 2 Medical Faculty Mannheim, Heidelberg University, Heidelberg, Germany; 3 Institute for Medical Technology of Heidelberg University and University of Applied Sciences, Mannheim, Germany; 4 Q-bios GmbH Biotechnology, Mannheim, Germany; Emory University School of Medicine, United States of America

## Abstract

The homodimeric transmembrane receptor endoglin (CD105) plays an important role in angiogenesis. This is highlighted by mutations in its gene, causing the vascular disorder HHT1. The main role of endoglin function has been assigned to the modulation of transforming growth factor β and bone morphogenetic protein signalling in endothelial cells. Nevertheless, other functions of endoglin have been revealed to be involved in different cellular functions and in other cell types than endothelial cells. Compared to the exploration of its natural function, little experimental data have been gathered about the mode of action of endoglin HHT mutations at the cellular level, especially missense mutations, and to what degree these might interfere with normal endoglin function. In this paper, we have used fluorescence-based microscopic techniques, such as bimolecular fluorescence complementation (BiFC), immunofluorescence staining with the endoglin specific monoclonal antibody SN6, and protein interaction studies by Förster Resonance Energy Transfer (FRET) to investigate the formation and cellular localisation of possible homo- and heterodimers composed of endoglin wild-type and endoglin missense mutant proteins. The results show that all of the investigated missense mutants dimerise with themselves, as well as with wild-type endoglin, and localise, depending on the position of the affected amino acid, either in the rough endoplasmic reticulum (rER) or in the plasma membrane of the cells. We show that the rER retained mutants reduce the amount of endogenous wild-type endoglin on the plasma membrane through interception in the rER when transiently or stably expressed in HMEC-1 endothelial cells. As a result of this, endoglin modulated TGF-β1 signal transduction is also abrogated, which is not due to TGF-β receptor ER trafficking interference. Protein interaction analyses by FRET show that rER located endoglin missense mutants do not perturb protein processing of other membrane receptors, such as TβRII, ALK5 or ALK1.

## Introduction

Endoglin (CD105) is a homodimeric transmembrane type-III co-receptor of the TGF-β signalling pathway [1] with a molecular weight of 180 kD [2]. It is highly expressed on proliferating endothelial cells. Mutations in the genes of endoglin or in the endothelial transmembrane receptor ALK1, a TGF-β type I receptor, cause the vascular disorder HHT (termed HHT-1 and HHT-2 accordingly) [3], [4]. The role of endoglin in HHT-1 has been further illustrated for endoglin heterozygous (+/−) knock-out mice [5], [6] that develop symptoms similar to those seen in humans, such as arteriovenous malformations (AVMs). Furthermore, the absolute importance of endoglin in angiogenesis has been demonstrated in double knock-out (−/−) mice, which die during embryogenesis around day 10, owing to developmental malfunctions of the vasculature [7]–[9].

Endoglin's function and its possible role in HHT was initially suspected [3] owing to its involvement in TGF-β signalling in endothelial cells [1], [10]. Subsequently, it was also reported that endoglin modulates BMP7, -9, and -10 signalling in endothelial cells too [11], [12]. Many aspects of the cellular mechanisms in which endoglin or HHT mutations play a role in TGF-β signalling remain not fully understood, as TGF-β induced cellular responses influenced by endoglin can be various and controversial [13], [14]. Moreover, steadily increasing experimental data reveal more and more functional aspects of endoglin apart from its sole involvement as a TGF-β or BMP signalling co-receptor. For example, endoglin influences the composition of focal adhesions [15], the organization of the cytoskeleton [16], interacts with the dynein light chain motor protein Tctex2β [17], and is involved in preeclampsia as a proteolytically cleaved extracellular soluble peptide [18], [19].

TGF-β signalling in endothelial cells is mediated by the TGF-β receptor complex in the cell membrane and specific members of the Smad protein family [20], [21], intracellular signalling mediators. The receptor complex can be composed of the main TGF-β type-II receptor TβRII and the type I receptor ALK5 or, with regard to HHT, of the type II receptor TβRII and the two type I receptors, ALK1 and ALK5 [22]. Upon TGF-β ligand binding, TβRII phosphorylates the type I receptor(s) that, in turn, phosphorylate receptor-specific Smads (R-Smads), mediating two different signalling cascades. The R-Smads 1 and 5 are activated by ALK1 and the R-Smads 2 and 3 by ALK5 [23], [24]. Subsequently, the phosphorylated R-Smads bind to another Smad family member, the common Smad4, and are then shuttled into the nucleus to regulate gene expression of various genes.

Endoglin was found to be associated with the TGF-β receptor complex [1], [25] and it has been shown to modulate the TGF-β signal between the ALK1 and ALK5 pathway in endothelial cells in favour of ALK1 [13], [26], [27], leading to opposite cellular responses between an activated state of cell proliferation and migration (for ALK1), and a quiescent state of the cells, with ALK5 mediated inhibition of both of the former, depending on the relative presence or absence of endoglin, a mechanism which has also been referred to as “angiogenic switch”, next to other forms of endothelial activation [28]. Moreover, endothelial TGF-β responses can vary strongly depending on the cytokine concentration, having more stimulating effects at low concentrations, while higher concentrations can have an opposite effect [23], [29].

To date, more than 300 different HHT1 mutations have been reported (www.arup.utah.edu/database/hht) that all affect the coding sequence of the extracellular domain of the endoglin protein. The majority of the mutations predict early protein sequence termination, owing to nucleotide insertions or deletions, which cause splice defects, frame shifts, or nonsense mutations, all leading to premature stop codons. Many of these mutations that have been analysed lead either to no detectable mutant mRNA [30], [31], or the mutant proteins seem to underlie, if expressed, such rapid degradation, that they cannot be detected, or only barely.

There is also a number of mutations that predict a full-length or near full-length protein sequence containing the extracellular, transmembrane, and intracellular regions of the protein. These are missense mutations, which contribute approximately 1/6 of all known mutations, and a few in-frame deletion mutations that are missing one or more amino acids in the extracellular domain. In contrast to the frame shift and nonsense mutations, protein expression could be confirmed for some of the missense mutant proteins, but these proteins were not detectable on the cell surface and it has been proposed that they resemble intracellular premature precursor proteins [32], [33].

It is assumed that haploinsufficiency is the underlying mechanism for HHT1, which means that only 50% of functional receptor protein is present at the cell surface. In several studies using isolated cells from HHT1 patients, such as HUVECs from newborns, activated monocytes, or blood outgrowth endothelial cells (BOECs), it was shown that these cells had reduced membrane surface expression levels of endoglin of about 40–50% [32]–[38], supporting the haploinsufficiency model. Beside the reduced endoglin levels in HHT1 patient samples, the question has been asked of whether missense mutant proteins have the ability to form heterodimers with wild-type endoglin and therefore might interfere with wild-type endoglin function [32], [33], [39], [40]. Recombinant expression of endoglin missense mutations revealed that these mutants are intracellularly retained and self-dimerise. Additional analyses by co-immuno precipitations demonstrated that the mutant proteins also dimerise with wild-type endoglin [40], therefore suggesting that they are capable of interfering with endoglin function. Furthermore, expression of a series of different, engineered, truncated extracellular forms of endoglin, similar to a number of mutant proteins, was found for self-dimerisation, and was found to be extracellularly secreted, but not or only weakly capable of dimerising with wild-type endoglin [33], [40], [41]. In summary, when comparing the different patient sample analyses and recombinant endoglin mutant protein expression studies, the evidence for the proteins of certain mutations becoming expressed and being stable, and the question of whether these are able to dimerise with wild-type endoglin, remains contradictory.

In this study, we analysed a series of endoglin missense mutant proteins in order to investigate expression and intracellular localisation of endoglin missense and wild-type proteins. For this purpose, we used different fluorescence-based microscopic approaches. In order to investigate the above-mentioned lack of detectability of several mutant proteins in patient samples, we tested a commonly used monoclonal antibody (SN6) for reactivity with fluorescence-tagged mutant proteins. To analyze the capability for dimerisation of mutant proteins with wild-type endoglin, we used bimolecular fluorescence complementation (BiFC). This method has widely been shown to be a powerful tool for visualization and measurement of various protein interactions, as well as protein dimerisation [42]. In order to determine the effect of heterodimerisation and intracellular retention of these proteins, we measured the relative amounts of endogenous wild-type (wt) endoglin in the plasma membrane of endothelial cells (HMEC-1) expressing mutant proteins. In addition, to study the effect of mutant proteins on TGF-β signalling, a signalling assay was performed in HMEC-1 cells expressing endoglin missense mutants. As another physiological test, we also investigated the influence of mutant proteins on cell proliferation in CHO-K1 cells. Furthermore, we applied microscopic FRET (Förster Resonance Energy Transfer) analyses to see whether intracellularly retained endoglin missense mutants might also interact with ALK1, ALK5, or TβRII, and therefore affect their processing to the cell surface.

## Results

The majority of the experiments performed in the here reported study were done in CHO cells and with endoglin-EYFP fusion proteins for fluorescence microscopy imaging and analyses. CHO cells were chosen because they are easy to transfect and are not reported to express endoglin. Therefore, CHO cells are ideal to analyse endoglin wild-type (wt) and mutant protein processing and trafficking in a cellular environment not obscured by an endogenous endoglin protein like in endothelial cells. Nevertheless, we performed a control experiment with an EYFP-tagged endoglin^wt^ protein to ensure that the EYFP fusion protein is regularly processed, transported and integrated into the plasma membrane. CHO cells were transfected with an endoglin^wt^-EYFP expression construct. After an expression time of 24 hours non-permeabilised cells were fixed and immunostained with the endoglin-specific monoclonal antibody SN6 (TRITC-labelled) in order to detect only the membrane present endoglin protein. The EYFP-tagged endoglin, showed regular membrane presence (see Supporting information, Figure S1), demonstrating that the EYFP tag does not obstruct endoglin processing and trafficking. This is important to know and allows us to draw conclusions how our findings might apply to other cell types like i.e. endothelial cells.

### 1. Localisation and colocalisation analyses of wild-type and mutant endoglin proteins expressed in CHO cells

We were interested in the cellular localisation of wild-type (wt) and mutant endoglin when co-expressed as they are naturally in endothelial cells. In order to simulate the *in vivo* situation, in which wild-type and mutant proteins are co-present, CHO cells were transfected with EYFP- and ECFP-tagged endoglin constructs, either alone or wt and mutant together. CHO cells were used, as they do not express endoglin endogenously to interfere with the ectopic endoglin proteins. Further on, it allows for analysing the effect when both protein populations are supposedly present in equal amounts by transfecting equal DNA amounts of the constructs. The investigated mutations G52V, W149C, A160N, and G413V had been published previously (www.arup.utah.edu/database/hht). The S480C mutation was identified in a Scottish HHT family (Dr. Jonathan Berg, personal communication). The R571H amino acid change was identified in a person from Morocco, who was treated for a single sporadic brain AVM (Dr. Jonathan Berg, personal communication). Whether this amino acid change is a cause of the BAVM or just a polymorphism is not known. However, in a reference panel of more than 200 chromosomes, the underlying nucleotide change was not detected, nor was it present in the 1000-genome data base (www.1000genome.org). Nevertheless, R571H might represent a rare polymorphism in the European Caucasian population or in the North African population. No panel for the latter population was available (Berg, personal communication). Out of curiosity, we included R571H in our analysis.

**Figure 1:**
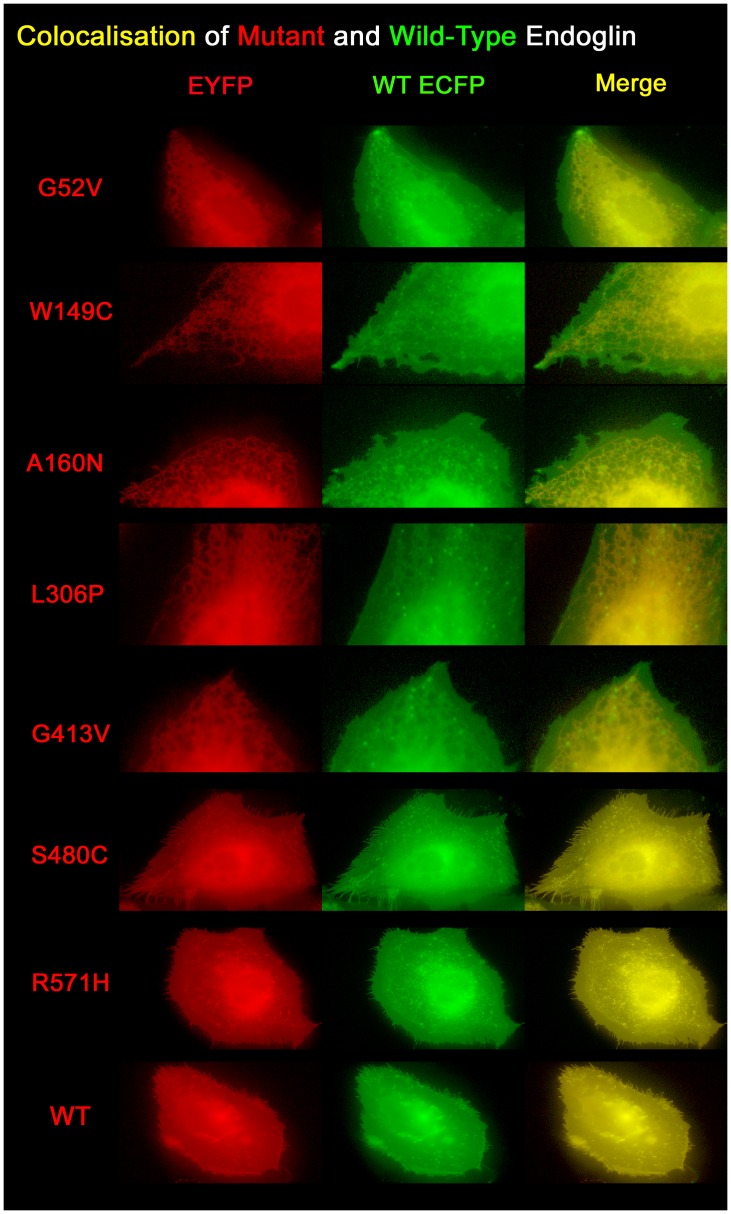
Wild-type and mutant endoglin proteins show intracellular colocalisation. CHO cells were co-transfected with equal amounts of endoglin^wt^ (ECFP-tagged) and endoglin missense mutant (EYFP-tagged) expression constructs as indicated. The localisation of expressed proteins was analysed by fluorescence microscopy. Irrespective of the fluorophore colours, mutant proteins are shown in red and endoglin^wt^ is shown in green. Mutants up to amino acid position 413 are retained inside the cell, while endoglin^wt^ shows membrane localisation, but also enhanced intracellular localisation when co-expressed with intracellularly retained mutants, as seen in yellow in the merged image.

In the experiments, cells were transfected with equal amounts of the respective constructs. In order to avoid experimental artefacts caused by high over-expression of endoglin proteins, only small amounts of plasmids were transfected (50 ng of each construct per 100 000 cells). When expressed on its own, wild-type endoglin (endoglin^wt^) shows typical plasma membrane localisation, while most of the missense mutants are trapped intracellularly, most likely in the rER, except S480C and R571H (Figure.1). Both proteins are expressed at the cell surface in the same way as endoglin^wt^. The G413V mutation appeared to be predominantly intracellularly expressed, but cell surface expression was occasionally also observed (single expression data not shown). However, in the co-expression experiments, cellular localisation of endoglin^wt^ changes in the presence of those mutants that remain intracellularly, sharing their fate of not being incorporated into the plasma membrane (Figure 1). In contrast, co-expression of endoglin^wt^ with endoglin^S480C^ or endoglin^R571H^ had no adverse effect on the previously observed cellular localisation of all three proteins. Co-expression studies of fluorescence-tagged endoglin^wt^ and endoglin mutants were performed with interchanged fluorophores. In one set of experiments endoglin^wt^ was ECFP-tagged and the mutants were EYFP-tagged (Figure 1). In a second set of experiments endoglin^wt^ was EYFP-tagged and the mutants were ECFP-tagged (Figure S2). However, this change of fluorophores had no influence on protein processing and trafficking as well as dimerisation. In both sets of experiments endoglin^wt^ strongly colocalised with the intracellular retained mutant proteins.

In order to have a measure for the colocalisation between the mutants and endoglin^wt^, we performed a Pearson correlation coefficient analysis. Colocalisation of endoglin^wt^ either with itself or with endoglin^S480C^ or endoglin^R571H^ resulted a Pearson correlation of ∼99% (Figure S3). Colocalisation of endoglin^wt^ with G52V, W149C, A160N, or G413V resulted a Pearson correlation of ∼90% for intracellular based colocalisation. In an additional analysis, which we conducted with rat microvascular endothelial cells (REC), we observed the same colocalisation patterns (Figure S4) as seen in CHO cells and measured comparable Pearson correlation values (Figure S3). This demonstrates that the results obtained for the endoglin mutants in CHO cells are comparable to those in endothelial cells. Pearson correlation coefficients were calculated for each combination from three independent experiments. Furthermore, we used the dopamine receptor DRD1 as a negative control in an additional experiment. DRD1, a single transmembrane receptor, is also expressed naturally in endothelial cells [43], but to the best of our knowledge does not interact with endoglin. Co-expression of DRD1 with endoglin^wt^ showed no strong colocalisation in the plasma membrane and co-expression of DRD1 with G52V showed no colocalisation at all with this intracellular retained mutant protein (Figure S5). This result supports the notion that the endoglin mutant proteins specifically obstruct endoglin^wt^ processing and trafficking.

### 2. Intracellularly retained endoglin missense mutants are retracted in the rough endoplasmic reticulum (rER)

**Figure 2:**
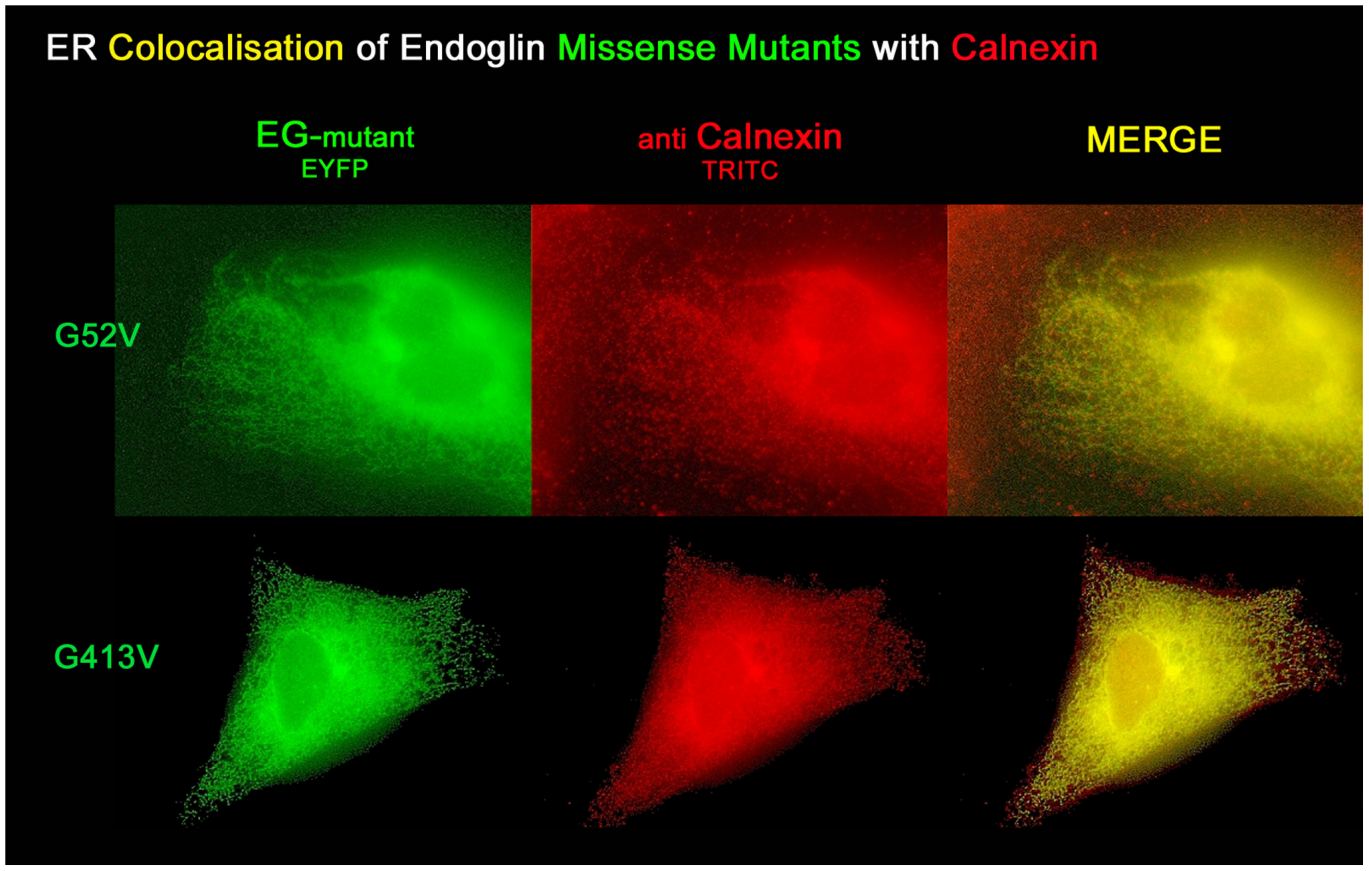
Endoglin missense mutants colocalise with the rER resident protein Calnexin. HMEC-1 cells were transfected with EYFP-tagged endoglin missense mutant constructs, permeabilized, and antibody stained against the rER resident chaperone protein calnexin. Two mutant proteins, G52V and G413V, were exemplarily investigated, with G52V resembling the lowest amino acid position and G413V representing the highest amino acid of the missense mutants here analysed that are intracellularly retained.

We suspected that the intracellularly localised mutants are retained in the rER. To confirm this hypothesis, we performed an immunostaining for the rER resident chaperone protein calnexin in cells transfected with endoglin mutant expression constructs. Microvascular HMEC-1 endothelial cells were transfected with EYFP-tagged mutant constructs, endoglin^G52V^ and endoglin^G413V^, that had shown suspected rER localisation. One day after transfection, cells were fixed and incubated with a monoclonal anti-Calnexin primary antibody and a TRITC-conjugated secondary antibody. Subsequent fluorescence microscopy analysis showed a definite colocalisation with Calnexin (Figure 2). This proves that those mutants that are not fully processed to the cell surface are indeed trapped in the rER, most likely not passing the protein quality control [44].

### 3. Endoglin missense mutants form homodimers and heterodimers with wild-type endoglin

Previous work has suggested that endoglin^wt^ and the endoglin mutant proteins are able to interact, as shown by co-immunoprecipitation analysis with mutants and a shortened endoglin^wt^ form expressed in COS cells [40]. Nevertheless, we wanted to test whether these results can be recapitulated by other, more “visual” techniques. Bimolecular fluorescence complementation (BiFC) is a powerful method for the investigation of protein interactions and receptor dimerisation [45], [46]. We therefore employed the BiFC method to investigate endoglin^wt^ and endoglin mutant protein interaction. For this purpose, EYFP-split expression vectors were engineered that contained the N-terminal part of the EYFP protein from amino acid position 1 to 172, named N172, and one construct with the C-terminal part of the EYFP protein from amino acid 173 to 238, named 173C, respectively. The results of our initial evaluation experiments have already been published [47] and show that, upon co-expression of the endoglin-split-EYFP fusion proteins endoglin^wt^-N172 and endoglin^wt^-173C in CHO cells, the split EYFP protein becomes reconstituted and its fluorescence restored. This demonstrates that this technique is well suited for endoglin protein interaction studies. In this previous study, we had also already started to test whether wild-type and mutant endoglin protein interactions can be investigated by the BiFC method. Here we present a more in-depth analysis.

**Figure 3:**
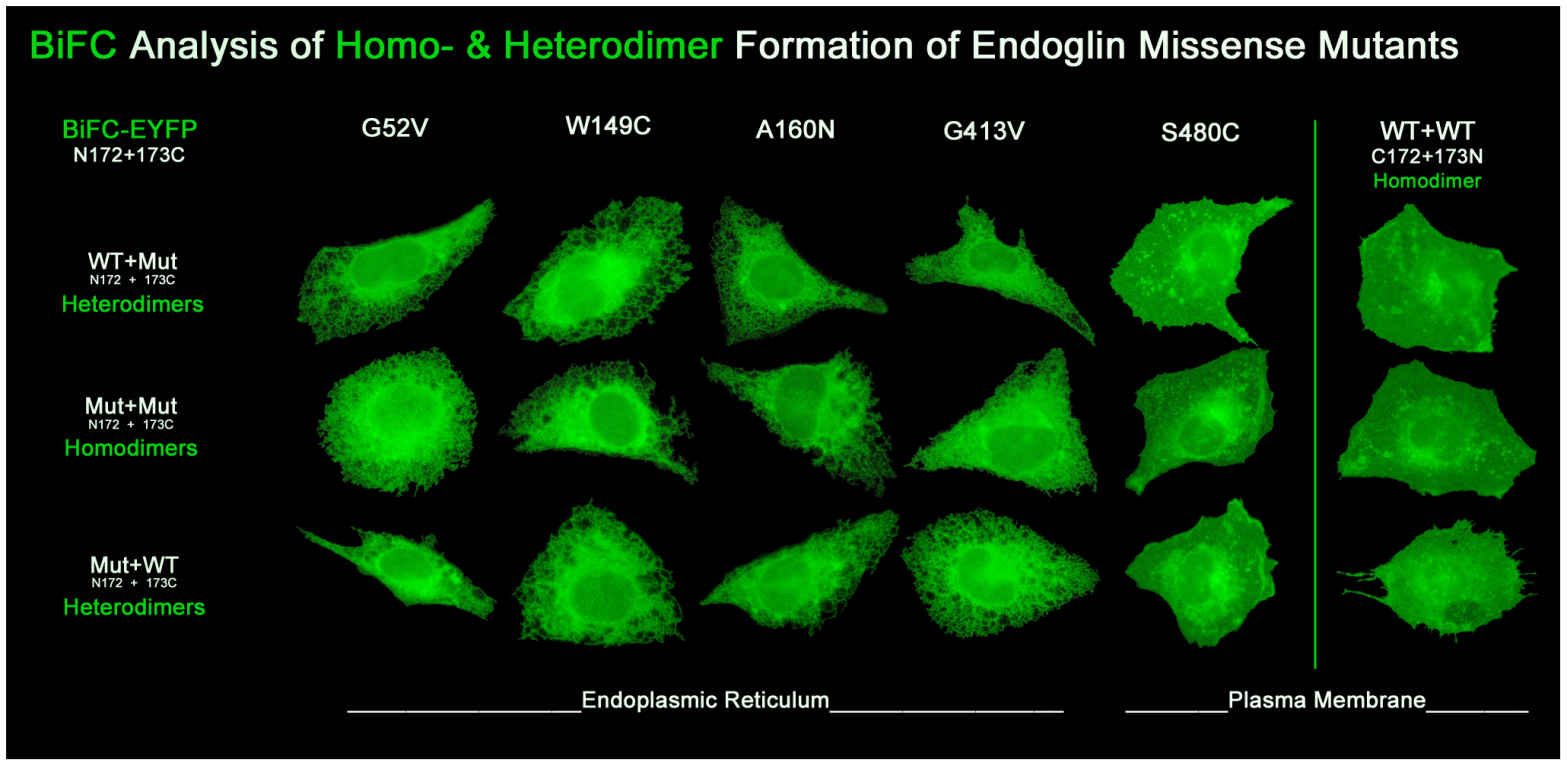
Endoglin missense mutants form homodimers and heterodimers with wild-type endoglin as demonstrated by BiFC. In order to investigate the possible assembly of mutant and wild-type (wt) homo- and heterodimers, the Bimolecular Fluorescence Complementation method was applied. The BiFC constructs represent the different endoglin protein variants to which either the N-terminal part of the EYFP protein, N172 (aa 1–172), or the C-terminal part of the EYFP protein, 173C (aa 173–238) was fused to the C-terminus of the respective endoglin variant. In the case of dimerization of an endoglin-N172 and endoglin-173C protein, the EYFP becomes recomplemented and EYFP fluorescence is restored. CHO cells were co-transfected with different endoglin BiFC construct combinations, as indicated. For the visualization of the heterodimers (row 1+3), both possible BiFC combinations are illustrated, in which the two BiFC fragments N172 and 173C fused to the two different endoglin variants were interchanged. The right column of the image shows only endoglin^wt/wt^ homodimers, as interchanging of BiFC fragments does not apply.

In order to test the different mutant proteins with or without endoglin^wt^, BiFC constructs were co-transfected in any possible combination of the dimerisation partners and their respective fused compatible EYFP fragments to selectively investigate the formation of mutant and wild-type homo- and heterodimers. The two different BiFC fragments (split EYFP fragments) can be interchanged within the interaction partners. All investigated mutants clearly showed the ability to dimerise with themselves and are retained intracellularly, as seen before with the EYFP-tagged proteins, except for endoglin^S480C^. This one is present at the cell surface like the wild-type protein (Figure 3). However, more importantly, the BiFC method confirmed a previous report [40] that all mutants dimerise with endoglin^wt^, as demonstrated by the restored fluorescence. Furthermore, endoglin^wt^, heterodimerised with the mutant, displays the same cellular localisation as the mutant proteins alone. This shows that the wild-type protein is trapped intracellularly, owing to the dimerisation with mutant endoglin.

In a flow-cytometry based control experiment we verified that the restored fluorescence of the BiFC fragments is specific due to endoglin dimerisation and is not the result of an auto-complementation by the BiFC fragments. Verification is based on the quantification of BiFC generated fluorescence (number of fluorescence events). For more details see supporting information Figure S6. A further result of this control experiment was that all of the mutants showed the same preference to dimerise with itself or with endoglin^wt^.

### 4. Missense mutant proteins force the wild-type endoglin protein into a mutant protein shape in heterodimeric complexes

**Figure 4:**
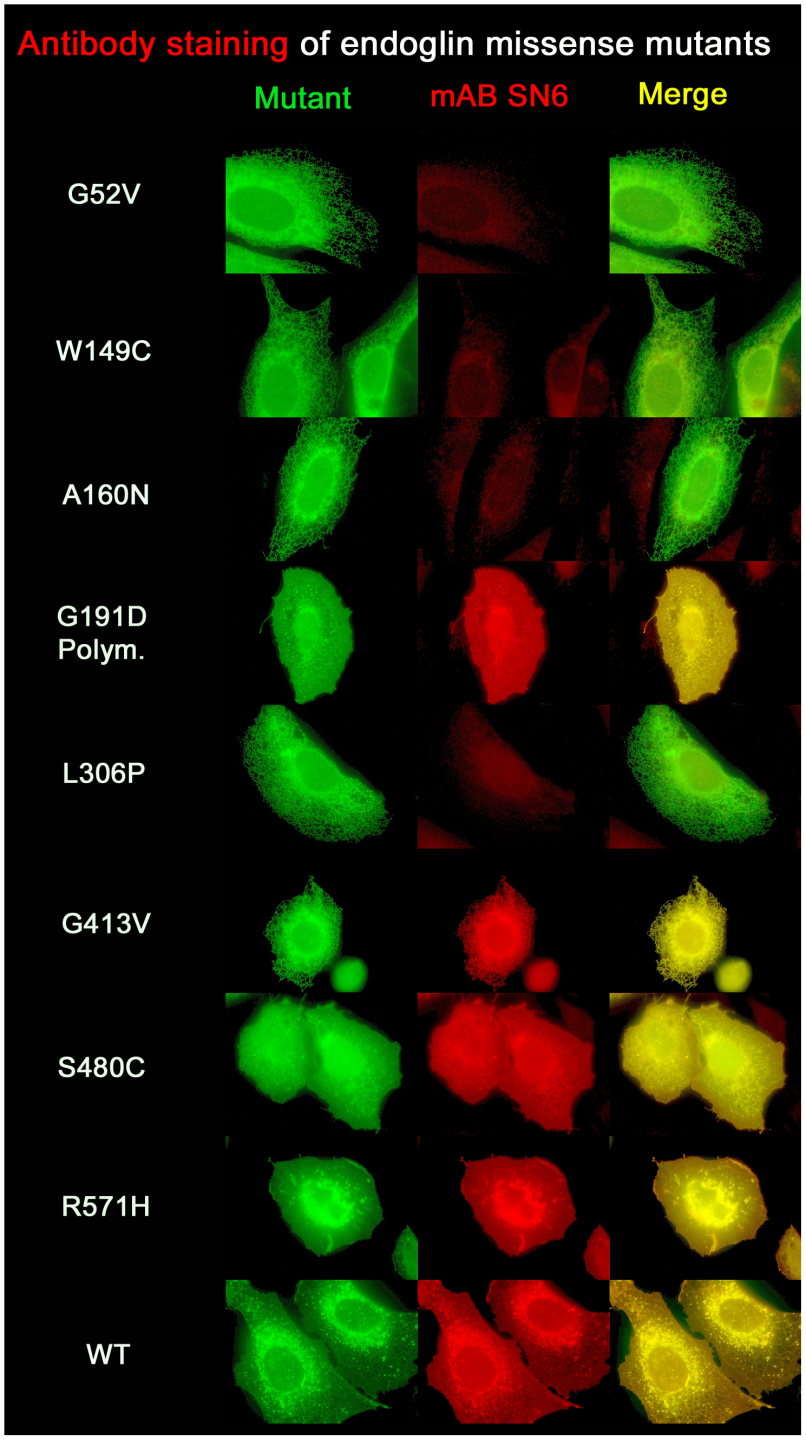
Detection of endoglin missense mutants with monoclonal antibody SN6 is epitope dependent. CHO cells were transfected with EYFP-tagged endoglin mutant constructs, permeabilized, and stained with the anti-endoglin antibody SN6, which binds to the amino acid epitope AS Y277-P338. Staining failed for all mutants with a missense mutation affecting amino acids 52, 149, 160 and 306, but was positive for the missense mutants G413V, S480C, and R571H, as well as for the endoglin polymorphism G191D.

In order to also investigate untagged mutant constructs for expression and localisation, we tried to detect mutant proteins in transfected CHO cells by immunostaining with the monoclonal anti-endoglin antibody SN6, which binds to the amino acid epitope AS Y277-P338 [48]. Staining with SN6 showed the typical cell surface distribution for endoglin^wt^, but failed for all mutants with rER localisation, except for mutation G413V (data not shown). The lack of staining might have been the result of poor transfection efficiency. We then turned to the EYFP-tagged mutant constructs, since successfully transfected cells should show green fluorescence, as was the case. Nevertheless, SN6 detection again failed for most of the mutants that were retained within the rER, except mutation G413V, as seen before with the non EYFP-tagged mutant protein. In addition, mutants S480C and R571H were readily detected, as was endoglin^wt^. The same applied to the known polymorphism G191D (Figure 4). These data suggest that the ability of the antibody to react with the mutants depends on the position of the mutation within the polypeptide strand in correlation to the position and relative distance of the antibody's binding epitope.

Our BiFC experiments demonstrate that binding of endoglin^wt^ to mutant endoglin occurs already in the rER, and thus endoglin^wt^ can also be trapped in the rER. This suggests that the mutant protein dictates the folding of the wild-type protein, becoming a quasi-mutant, which, in this heterodimeric complex, also does not pass the rER quality control. Consequently, SN6 staining of wild-type/mutant heterodimers is also expected to fail. To prove this hypothesis and to be sure that heterodimers are definitely present, we used the BiFC constructs again. CHO cells were co-transfected with endoglin^wt^-N172 and mutant-173C constructs, thus to be able to exclusively visualize the heterodimers, owing to EYFP fluorescence restoration, and cells were then stained with the SN6 antibody.

**Figure 5:**
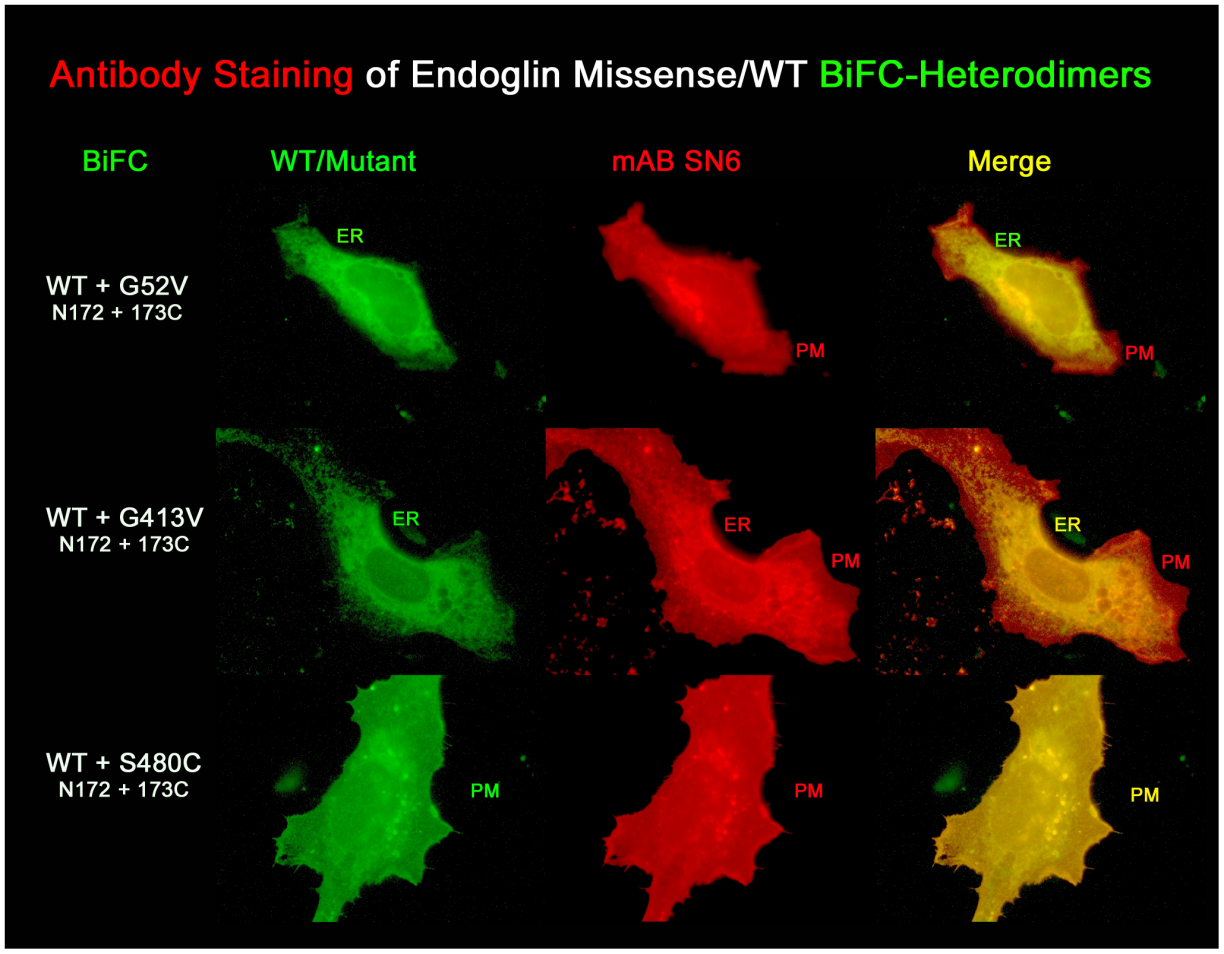
Detection of G52V homodimers as well as G52V/wild-type heterodimers failed with the monoclonal antibody SN6. In order to selectively visualize mutant/wt heterodimers, CHO cells were co-transfected with endoglin EYFP-BiFC constructs, as indicated. Cells were permeabilized and stained with the endoglin-specific monoclonal antibody (mAb) SN6 (red fluorescence) and analysed for colocalization by fluorescence microscopy. In this experiment, endoglin wild-type homodimers, as well as mutant homodimers are formed, but are not visible by green fluorescence, since recomplementation of EYFP occurs only within wt/mutant heterodimers. The G52V/wt heterodimer BiFC complex is found only in the rER. As shown in the merged image, after antibody staining this compartment shows a more greenish colour, rather than a complete yellow/orange colour, which would be the case if the antibody were to bind to the rER localised heterodimer. The incomplete green colour of the rER after SN6 staining is most likely the result of antibody detectable premature endoglin^wt^ homodimers in the rER. In contrast, the rER retained G413V/wt heterodimer is detected by the antibody in the rER, as seen in the merged image (rER appears in yellow). Membrane (PM) localized S480C homo- and S480C/wild-type heterodimers are also readily detected by the SN6 antibody.

As a result, detection of the BiFC heterodimers by SN6 failed in the same manner as before for the mutant homodimers (Figure 5), thus proving our hypothesis that the overall shape of the wild-type/mutant heterodimer molecule becomes misfolded in such a way that even the epitope of the wild-type protein is masked. Nevertheless, in the wild-type and mutant co-transfection experiments, a weak cell surface staining was observed. This is because three different dimers can be formed, wt/wt, mutant/mutant, and wt/mutant. Therefore, a cell membrane staining, but no intracellular staining, was observed, for example, in endoglin^wt^ and endoglin^G52V^ co-transfected cells, whereas endoglin^wt^ and endoglin^G413V^ co-transfected cells showed cell membrane staining, as well as intracellular staining of the BiFC dimers formed. In the case of the combination of endoglin^wt^ and endoglin^S480C^, full membrane staining was observed. However, it becomes difficult to distinguish between endoglin homo- and heterodimers, as both protein types localise in the same compartment. But it can be assumed that the antibody does recognize the heterodimer in the same way as was the case for the mutant S480C homodimers.

### 5. Endoglin missense proteins reduce membrane presence of the endogenous wild-type endoglin protein in HMEC-1 endothelial cells

To further investigate the scavenging effect of the mutants on the wild-type endoglin protein, we were interested to see to what extent endogenous wild-type endoglin would be reduced on the cell surface when held back in the rER through mutant heterodimerisation. HMEC-1 endothelial cells were thus transfected with the different EYFP-tagged mutants, so as to be able to discriminate transfected from non-transfected cells. Three different mutants were chosen: firstly, G52V with the most N-terminal mutational sites available; secondly, G413V, the first mutation that can be detected with antibody SN6; and lastly, A160N as a representative of mutations in between the two. In addition, the dopamine receptor DRD1 was used as a negative control, to compare any interfering effects of ectopic protein expression on endogenous protein synthesis.

**Figure 6:**
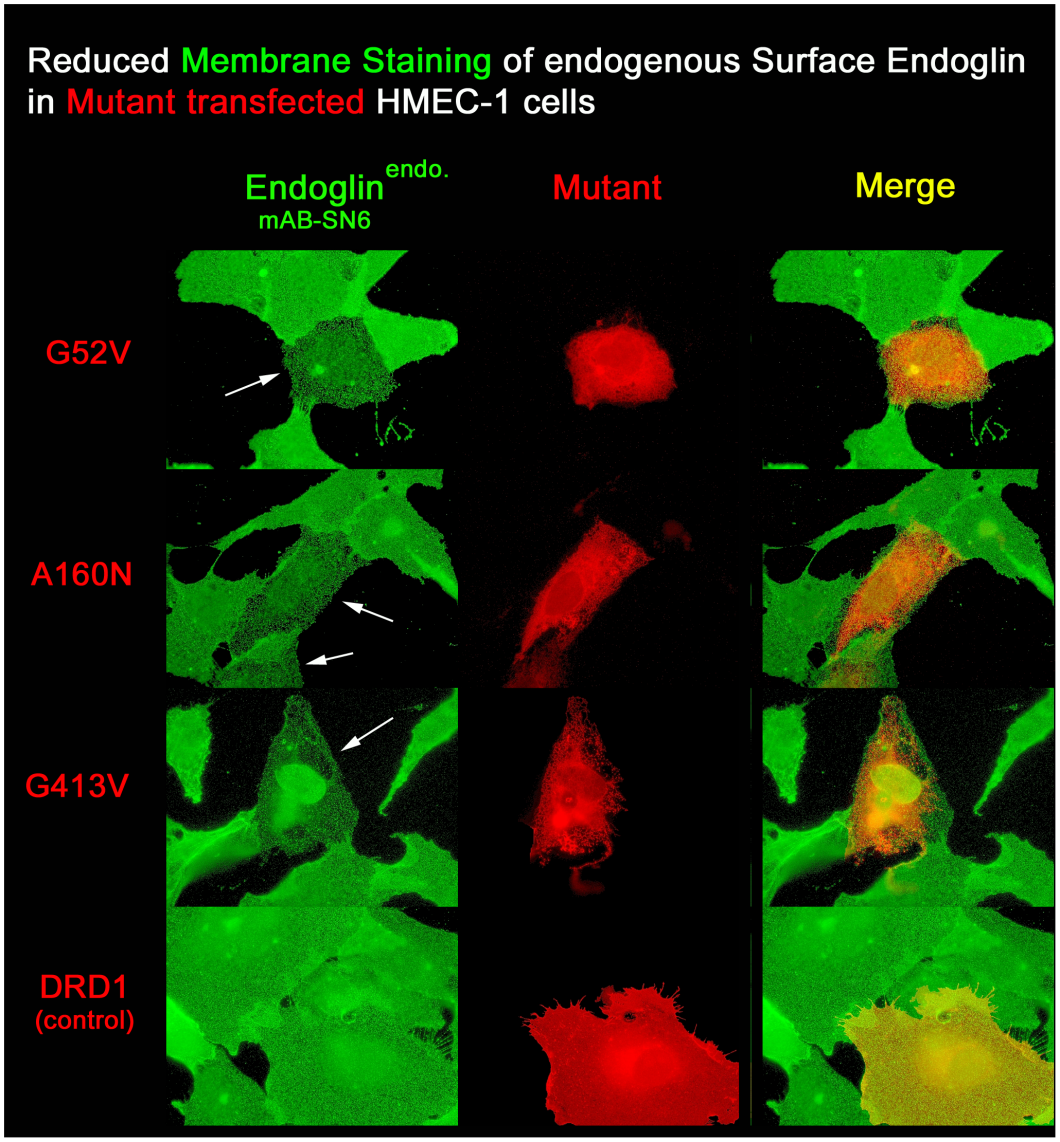
Ectopic expression of rER located missense mutants interferes with membrane processing of endogenous endoglin in HMEC-1 cells. In order to analyse whether endoglin missense mutants might interfere with the processing of endogenous endoglin (Endoglin^endo^) or not, HMEC-1 endothelial cells were transfected with EYFP-tagged mutants or the EYFP-tagged DRD1 receptor, as indicated (all false-coloured in red). The membrane localized DRD1 receptor, not known to interact with endoglin, served as a negative control. After 24 hours, non-permeabilized cells were immunostained with the endoglin-specific monoclonal antibody (mAb) SN6. Non-permeabilization allows endogenous endoglin only to be detected in the membrane (shown in green). Cells transfected with the endoglin missense mutants (indicated by arrows) show a clear reduction of green fluorescence for the endogenous endoglin in the membrane in comparison to adjacent non-transfected cells. Ectopic expression of the DRD1 receptor appeared to have little or no effect on the membrane presence of endogenous endoglin.

**Figure 7:**
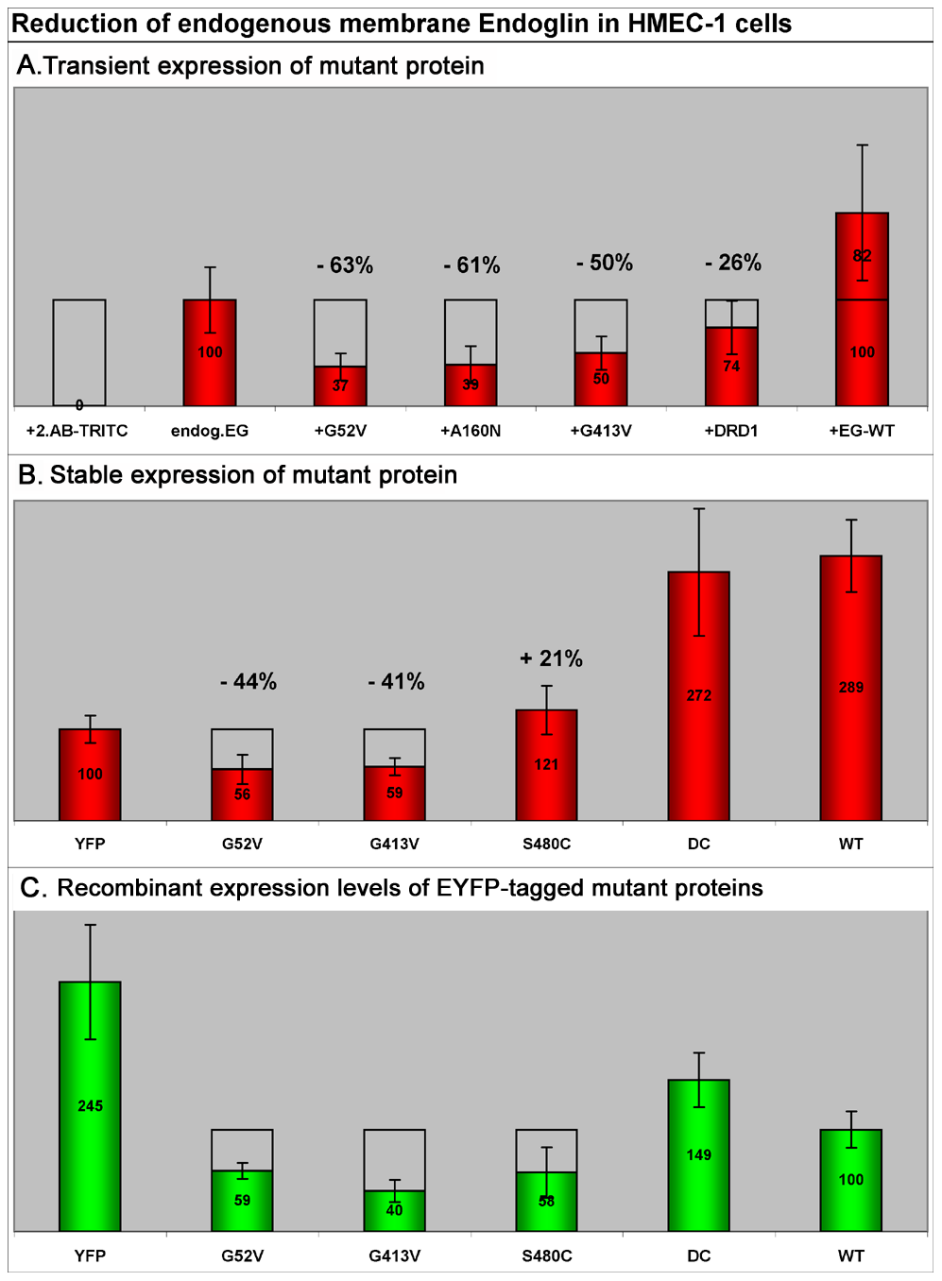
Relative membrane levels of endogenous endoglin in HMEC-1 cells upon transient or stable mutant protein expression. For quantitative measurements of endogenous surface endoglin, cells were fixed, immunostained with mAB SN6 (TRITC), and analysed by fluorescence microscopy. To exclusively stain surface endoglin, the cells were not permeabilized. **A.** Relative quantification of membrane endoglin after 24 hours of transient expression of mutants and the DRD1 receptor. In comparison to non-transfected HMEC-1 control cells, mutant expression resulted in a total reduction of endogenous membrane endoglin by 50–60%. Expression of the DRD1 receptor decreased the membrane endoglin level by 26%. **B.** HMEC-1 cells were stably transfected with different EYFP-tagged endoglin mutants and for control comparison also with endoglin^wt^, endoglin^ΔC^ (DC, without cytoplasmic domain), and just EYFP. Stabilized cells were FACS sorted for selection of endoglin variant expressing cells. Subsequently, cells were fixed, immunostained with mAB SN6, and levels of surface endoglin were measured by fluorescence microscopy. In comparison to only EYFP expressing control cells, the rER trapped missense mutants G52V and G413V reduced membrane levels of endogenous endoglin by ∼40%. Expression of the membrane localized mutant S480C raised the level of SN6-detectable molecules in the membrane by 21%. In contrast, additional expression of endoglin^wt^-EYFP and endoglin^ΔC^-EYFP raised the membrane levels of SN6-detectable molecules by ∼180%. **C.** Expression levels of EYFP-tagged stably expressed recombinant proteins. Values were normalized to the amount of recombinant expressed endoglin^wt^ (100%). The amount of the different endoglin mutants was only ∼55% of that of endoglin^wt^. During cell stabilization, only comparably low expression levels of all missense proteins seem to have been tolerated. Cells expressing the variant endoglin^ΔC^ (DC, without cytoplasmic domain) showed a 1.5 times higher ectopic expression than endoglin^wt^.

Twenty-four hours after transfection, non-permeabilized cells were stained with the SN6 antibody against wild-type endoglin present on the cell surface and fluorescence intensities were measured. Within the transient transfection experiments, non-transfected EYFP-fluorescence negative cells served as an internal control and reference for the normal cell surface presence of the endogenous endoglin protein. As a result, all of the tested mutants caused a clear reduction in the amount of endogenous wild-type endoglin on the surface of endothelial cells (Figure 6). The ectopic expression of the DRD1 receptor led to a slight reduction of membrane endoglin, and in multiple cases to none, and was far from being as dramatic as observed for the endoglin mutants. The background-corrected intensity measurements showed a mean endoglin surface reduction of 26% for the DRD1 receptor expressing cells, compared to the non-transfected control samples. This might suggest a possible general interference of ectopic protein expression with endogenous protein production and processing, since DRD1 is fully processed to a mature glycosylated 7-transmembrane protein of 50 kD, in contrast to the endoglin mutants which reside in the ER. The mutant proteins reduced the amount of surface endoglin by 63% (G52V), 61% (A160N), and 50% (G413V), respectively, and by 37% (G52V), 35% (A160N), and 24% (G413V), respectively, when corrected for the endogenous endoglin surface reduction caused by DRD1 (Figure 7.A).

The previous experiment investigated the effect of transiently expressed missense mutants on endogenous wild-type endoglin cell surface levels. The reduction of endogenous endoglin in these experiments may have depended on the transfection efficiency for the missense mutant constructs, which may not necessarily represent the physiological situation. Therefore, we stabilized and FACS sorted HMEC-1 cells transfected with an EYFP expression vector and different EYFP-tagged wild-type and mutant endoglin constructs, in order to analyse the amount of membrane-localised endogenous wild-type endoglin after adaptation to the mutant proteins. In the following experiments, the EYFP stabilised HMEC-1 cells served as the reference cell line when analysing the effect of mutant proteins on endogenous endoglin in HMEC-1 cells.

Instead of A160N, the mutation S480C was used as a further control, because this mutant is translocated to the membrane, can be detected with the SN6 antibody, and therefore should result in an increased membrane-localised amount of SN6-detectable endoglin proteins. In addition, we were further interested to see what effect the lack of the endoglin intracellular region might have on processing and membrane localisation. This endoglin variant has been described previously and is called delta-cyto (ΔC) [40].

Stable expression of the two rER-retained mutant proteins G52V and G413V in HMEC-1 cells led to reduced membrane levels of the endogenous endoglin by about 40%. Surprisingly, stable expression of the S480C mutant increased the amount of SN6-detectable membrane endoglin by only 20%, whereas cells expressing wild-type endoglin or the ΔC variant showed increased levels of 190% and 170%, respectively (Figure 7.B). Interestingly, expression levels of the mutant proteins were comparable between the two intracellularly retained mutations G52V and G413V and the membrane localised mutant S480C, as measured by their relative EYFP fluorescence intensities (Figure 7.C). In comparison, the expression levels of ectopic wild-type endoglin and the ΔC variant were approximately two times higher than those for the missense mutant proteins. This might suggest that the cells do not tolerate the presence of the mutant proteins well, and may have regulated their expression down to a level that is still tolerable for the cells.

### 6. Endoglin's inhibitory activity on the TGF-β1-ALK5-Smad3 signalling pathway in endothelial cells is abrogated by endoglin missense mutant proteins

The previous experiments demonstrated that the missense mutant proteins are highly capable of forming dimers with endoglin^wt^, which leads to a reduction of wild-type endoglin at the cell surface in combination with most of the mutants. We were therefore interested in what consequence this might have for the modulating role of endoglin in TGF-β signalling in endothelial cells. Here, we were especially interested in the Smad3 pathway, since it is known that endoglin has an inhibitory function in this signalling route [13], [27]. For this purpose, a TGF-β1 regulated signal assay was conducted in HMEC-1 endothelial cells and a rat endothelial cell line (REC), using the (CAGA)_12_-luciferase promoter reporter activated by the ALK5-Smad3 pathway. The capability of dimerisation between human and mouse endoglin had previously been demonstrated [41] and we therefore assumed that endoglin from another rodent species would also interact with human endoglin. We were curious to see whether a possible interaction between human endoglin mutants and endoglin^wt^ from a different species might also interfere with signalling.

**Figure 8:**
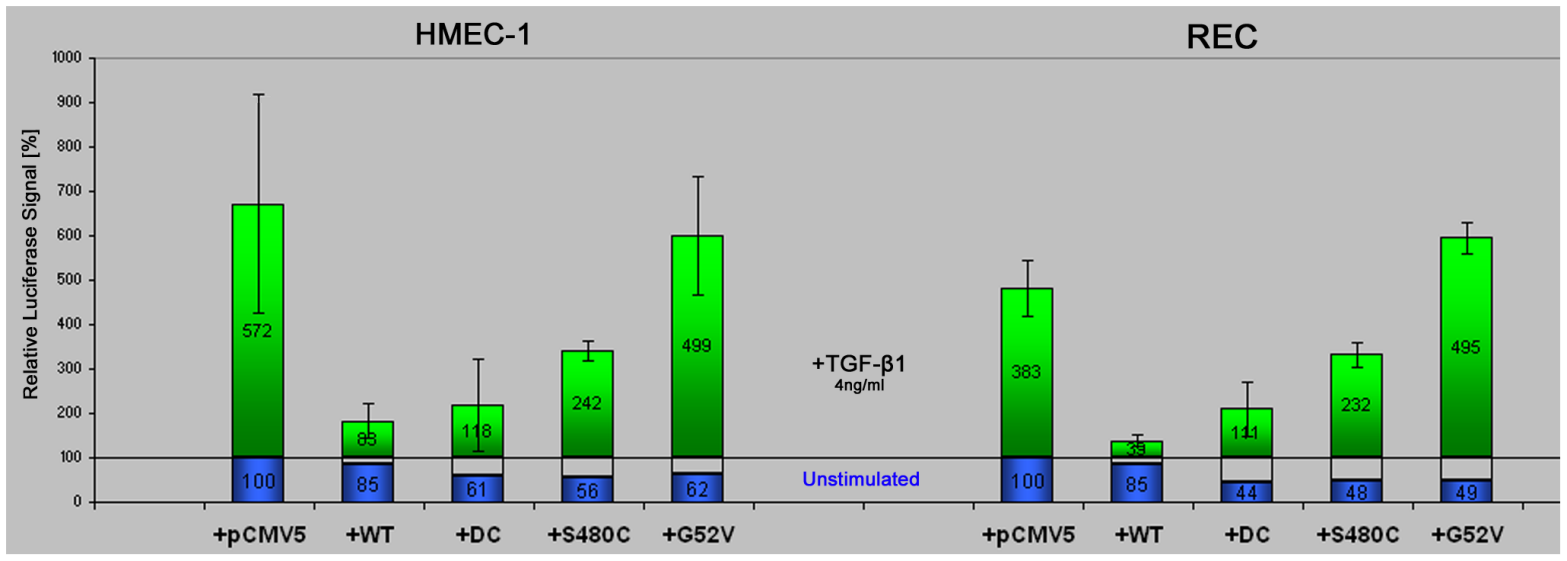
Endoglin missense mutants interfere with endoglin's inhibitory activity on TGF-β1 ALK5 signalling. HMEC-1 and REC endothelial cells were co-transfected with different untagged endoglin constructs, or empty vector (pCMV5), and the CAGA reporter plasmid as indicated. After an overnight stimulation with 4 ng/ml TGF-β1, cells were lysed and luciferase activity was measured. Values are given as a relative increase induced by TGF-β1 (upper column) over 2% FCS basal reporter activity of the individual non-stimulated control (lower column), and normalized to 100% basal activity of mock transfected cells. As shown, over-expression of endoglin^wt^ or endoglin^ΔC^ (DC, lacking the cytoplasmic domain) strongly reduced the TGF-β1 induced reporter activity. In contrast, mutant G52V expressing cells showed no reduction of the reporter signal, whereas expression of the plasma membrane localized mutant S480C reduced the signal by about 50%. Average values of two independent experiments are shown.

For the signalling assay, cells were transfected with the reporter in combination with different endoglin expression constructs (endoglin^wt^, endoglin^ΔC^, endoglin^G52V^ and endoglin endoglin^S480C^) or an empty expression vector, and 24 hours later cells were incubated over night, with or without TGF-β1. Subsequently, cells were lysed and luciferase activity measured. The results obtained in both endothelial cell types were comparable, as seen in figure 8. Over-expression of wild-type endoglin substantially reduced the reporter activity as compared to mock transfected cells, which is in accordance with previous findings [13], [27]. Endoglin^ΔC^ (lacking the cytoplasmic domain) also led to a substantial inhibition of the ALK5 mediated reporter signal.

In contrast, no inhibitory effect was seen for cells transfected with the G52V mutant. In these cells, reporter activity was comparable to that of mock transfected cells or even increased, as observed for the rat endothelial cells. This suggests that intracellular interception of endogenous endoglin^wt^ by the intracellularly retained mutant G52V leads to a reduction of endogenous wild-type endoglin on the membrane surface and therefore to less endoglin capable of interfering with ALK5 signalling.

Interestingly, cells over-expressing the membrane-localised S480C mutant showed only some inhibitory activity towards TGF-β1 signalling, which was about half of that of endoglin^wt^ over-expressing cells. As shown before, S480C forms dimers with endoglin^wt^ at the cell surface. This might indicate a reduction of functionality of the wild-type/mutant heterodimer at the plasma-membrane, or that the mutant homodimer is not functional in this signalling assay, or both.

### 7. Influence of wild-type endoglin and missense mutants on cell proliferation in CHO cells

The pro-proliferative influence of wild-type endoglin has been repeatedly reported for endothelial, as well as non-endothelial, cell types [13], [49]–[51]. We were thus interested in discovering to what extent endoglin missense mutant proteins might affect cell proliferation in a TGF-β1-independent manner.

**Figure 9:**
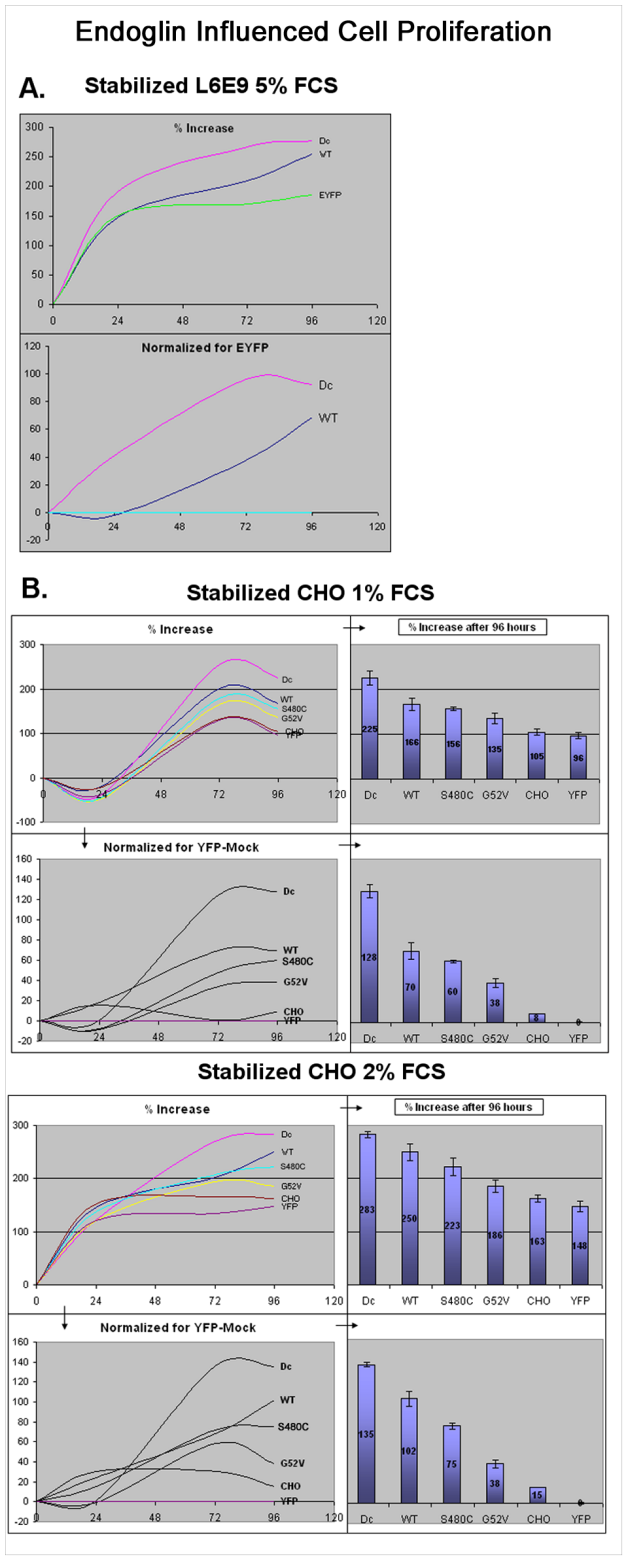
Endoglin^wt^ enhances cell proliferation in a TGF-β1 independent manner. L6E9 rat myoblasts and CHO-K1 cells were stabilized with different EYFP-tagged endoglin constructs or with empty vector EYFP-N1 (reference cells) as indicated. **A.** Proliferation of L6E9 rat myoblasts, cultured in growth medium supplemented with 5% FCS, was measured over a period of 96 hours. **B.** Proliferation of CHO-K1 cells was measured over a period of 96 hours in growth medium supplemented with 1% or 2% FCS. Proliferation values were compared to EYFP-N1 mock transfected cells. Proliferation rates of non-transfected CHO cells are also shown. Each time point represents the mean of 10 wells in a 96-well plate for the CHO cells; the time points for L6E9 cells are mean values of 20 wells per plate. Shown are the results of a representative experiment.

We first tested the effect on proliferation with L6E9 rat myoblast cell lines, stabilised for expressing endoglin^wt^-EYFP, endoglin^ΔC^-EYFP or just EYFP (Figure 9A). Endoglin^wt^ expressing L6E9 cells displayed a 60–70% cell number increase after 96 hours compared to EYFP expressing cells, confirming the previously reported general pro-proliferative effect of endoglin. Surprisingly, the expression of endoglin^ΔC^ led to an even greater increase of cell numbers of about 90%. This effect was also seen in the following experiments with stabilised Chinese hamster ovary (CHO) cells. The ΔC variant lacks the cytosolic domain, but otherwise shows normal cell surface localisation as previously reported [40]. Next, we tested what effect endoglin missense mutant proteins might have on cell proliferation. For this purpose, we generated a number of CHO cell lines stably expressing different EYFP-tagged endoglin variants, including endoglin^wt^, endoglin^G52V^ (rER localisation), endoglin^S480C^ (membrane localisation), endoglin^ΔC^, and the expression vector EYFP-N1, respectively. Stable transfected cells were expanded and green fluorescent cells were sorted for by fluorescence-activated cell sorting (FACS). The FACS sorted cell lines, representing polyclonal cell lines, were then used for further proliferation experiments. During our initial experiments, we observed that high FCS concentrations of 5% and 10% mask any transgenic effects on proliferation, so that differences were not detectable (data not shown). Therefore, in order to analyse the effect of the endoglin variants on cell proliferation, equal amounts of cells for each of the different cell lines were seeded in growth medium supplemented with low FCS concentrations of just 1% and 2%, respectively (Figure 9B).

1% FCS led to an initial population decrease, after which the cells recovered and started to proliferate. No cell number decline was observed for cells seeded in growth medium with 2% of FCS. The greatest increase of cell numbers was always observed after 72 hours, followed by a decline, as can be seen for 96 hours. The highest proliferation rate was consistently seen for the endoglin^ΔC^ cells, followed by endoglin^wt^ expressing cells. To our surprise, the missense mutant protein expressing cells also displayed a higher rate of proliferation than non-stabilized/non-EYFP expressing CHOs or the EYFP stabilized control cells, but had slower growth rates than the ΔC or wild-type endoglin cell lines. Among the different endoglin variants, the G52V expressing cells had the lowest proliferation rate, which was followed by cells expressing mutant S480C, wild-type endoglin, and then ΔC. Results are shown in detail in Figure 9B. Our observations with regard to the ΔC variant may suggest that the extracellular region of endoglin is responsible for the enhanced proliferation, which might be moderately regulated by the cytoplasmic domain.

### 8. Endoglin missense mutant protein G52V does not interfere in the rER with ALK1, ALK5 or TβRII

The previous experiments showed that the mutant endoglin proteins dimerize with endoglin^wt^ and therefore may interfere with the inhibitory activity of endoglin on the TGF-β1 induced ALK5-Smad3 pathway in endothelial cells. In the proliferation experiments, stable expression of endoglin^wt^ led to cell proliferation enhancement. Surprisingly, the mutants also increased the proliferation rate of CHO cells compared to EYFP-transfected CHO cells and non-transfected CHO cells, although not to the extent that endoglin^wt^ does. The proliferation results with the mutants seemed to be contradictory to the pro-proliferative effect of wild-type endoglin. ALK5 and TβRII are known to mediate the TGF-β1 induced inhibition of proliferation. We hypothesized that the intracellularly retained mutants may also interfere with ALK5 and TβRII processing, leading to a reduced amount of these receptors at the cell membrane, which in turn could cause the increased proliferation rate. In order to test our hypothesis of potential interactions of the receptors with endoglin missense mutants, we performed a Förster Resonance Energy Transfer (FRET) analysis using ECFP as donor and EYFP as acceptor tags. FRET is a technique that allows for quantitative measurement of protein interactions. The FRET effect takes place within a maximal distance of approximately 10 nm between the donor and the acceptor molecule. The amount of transferred energy ( =  FRET-efficiency) increases exponentially with decreasing distance of the two molecules, while it immediately drops to zero when the distance becomes greater than 10 nm. Therefore, the FRET-efficiency provides an indirect measure when the fluorophores are fused to two interacting proteins.

In the following experiments, we representatively tested the EYFP-tagged endoglin missense mutants, G52V and S480C, and the respective ECFP-tagged membrane receptors ALK1, ALK5 or TβRII in CHO cells. As a control for comparison, the receptors were also co-expressed with EYFP-tagged endoglin^wt^ to observe the normal sites of colocalisation and the degree of interaction. Furthermore, ECFP-tagged endoglin^wt^ and EYFP-tagged endoglin^G52V^ or EYFP-tagged endoglin^S480C^, respectively, were co-expressed to measure the formation of heterodimers in the rER, as well as in the membrane. As a further control, we used the ECFP-tagged DRD1 receptor to investigate a possible general negative effect of the endoglin mutants on protein processing in the ER. Additionally, we co-transfected integrin-alpha-6 (IA6) -ECFP together with endoglin^wt^ -EYFP to obtain a measure of possible FRET for two membrane proteins that to our knowledge are not reported to interact, therefore serving as a further negative control. Cytoplasmic localised soluble ECFP co-expressed with EYFP-tagged receptor proteins (ALK1, Alk5, TβRII, endoglin) served as a general negative control for FRET. The FRET index (FI) calculated from these experiments (see below) served as our FRET negative reference index. As a positive control for FRET in general, we used a cytoplasmic located EYFP-28 amino acid linker-ECFP fusion protein. This calibration probe produces intra- as well as intermolecular FRET. After calibration, the calculated FRET efficiency was 18% for our FRET-probe with our microscope system. This has to be considered the optimal efficiency in our system when compared to the following FRET efficiencies for endoglin/endoglin or endoglin/TGF-β receptor interaction analyses. Co-transfection of endoglin-EYFP and endoglin-ECFP resulted in efficiencies of 2–8% for endoglin homodimers (data not shown). In order to minimize over-expression artefacts, only 50 ng of each expression construct (100 ng in total) were used for co-transfections of 100 000 cells. Fluorescence microscopy pictures from live cell imaging were used for FRET calculations. The reported FRET data and analyses are the result of at least three independent experiments.

#### Classification of FRET indices

When measuring FRET efficiency images, we found that it was important to also take the respective area into account where FRET actually occurs in relation to the area where it is possible for FRET to occur (100%). This area is determined by the simultaneous presence of donor and acceptor molecules in the same image pixel. In order to grade the possible receptor interactions measured by FRET, a FRET index (FI) was calculated by multiplying FRET-efficiency with the area where FRET was measured. Therefore, even when the FRET-efficiency appears to be relatively low, i.e. 2.5%, but with a high abundance, e.g. on 100% of the possible area where FRET can occur, this results in a FRET index of 250. In contrast, where there are a few artefact pixels with very high FRET efficiency values, of 10% or more, but covering only an area of 2% (in regard of where FRET can occur), this results in a low FRET index of 20.

**Figure 10:**
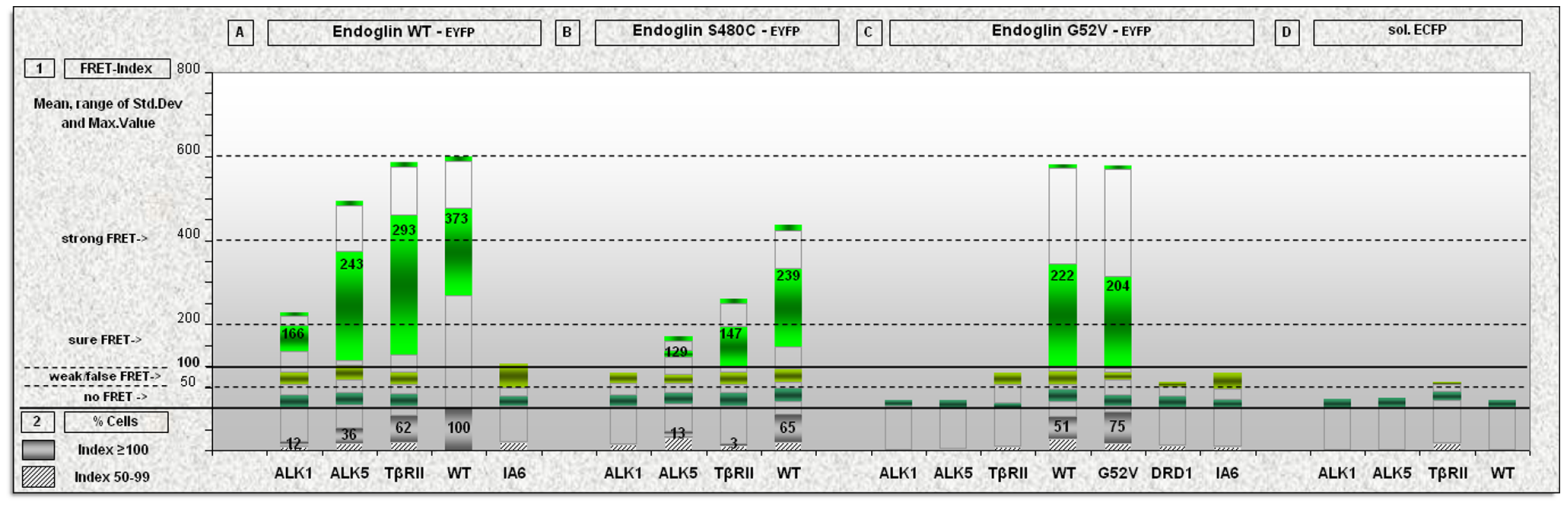
FRET analysed degree of protein interactions between endoglin^wt^, mutant S480C, mutant G52V, and TGF-β receptors ALK1, ALK5, and TβRII. CHO cells were transfected with the different ECFP-tagged membrane receptors and co-transfected either with **A.** endoglin wild-type (EYFP-tagged), **B.** with the mutant S480C (EYFP-tagged), or **C.** with the mutant G52V (EYFP-tagged). **D.** Soluble cytoplasmic ECFP co-transfected with EYFP-tagged TGF-β receptors served as a general negative control. Cells were seeded in glass dishes suited to fluorescence microscopy and analysed by live cell imaging 24 hours after transfection. A minimum of 30 cells for each analysed protein interaction was evaluated to determine FRET indices. The diagramme summarises the results for each receptor combination. (**1**) Upper part of the diagramme: based on the negative controls, FRET index values were sorted into three groups: Group1: FI<51 (no interaction), Group2: FI 51–99 (no/weak interaction or false positive), Group3: FI≥100 (interactions of different strengths). Distribution of the FRET-index values within the groups is shown within the standard deviation. The mean value is indicated for Group 1 and the maximum is shown graphically. (**2**) Lower part of the diagramme: frequencies (number of cells in %) of FRET occurrences for the positive group (grey bar) and for the second group (FI 51–99, hatched bar). The value is given for the positive group. Frequencies of Group 3 (FIs<50) are not shown.

In order to decide for the different receptor combinations whether a measured FRET index represents a positive protein interaction or not, we first calculated the FRET indices of the negative controls (Figure 10.D), consisting of soluble cytoplasmic ECFP in combination with the EYFP-tagged receptors. The measured FRET indices of the controls were between 0 and 50, except for TΒRII, which also had values slightly above 50. As an additional control for a rER processed membrane receptor protein we used integrin-alpha-6, which is to the best of our knowledge not known to interact with endoglin. Cells co-transfected with these two proteins reached maximal FRET index values of approximately 100. Therefore, we set the minimum limit for the positives to a FRET-index of 100. Nevertheless, values between 50 and 100 may still represent weak or rarely occurring positive protein interaction events. Based on this, FRET indices were grouped as follows: Group 1: FI<51 =  no protein interaction; Group 2: FI between 51 and 99 =  weak or false positive protein interaction; Group 3: FI≥100 =  indicating protein interaction. Finally, in order to compare the degrees of interaction of the different receptor combinations, we used the standard deviation of the positive group to show the distribution of the FRET-index values. As a second measure, the frequencies (% of cells showing FRET) within the groups were determined.

#### Results of the FRET analyses

First we analysed the protein interactions in the membrane between endoglin^wt^ and ALK1, ALK5, and TβRII, respectively, as well as the formation of endoglin^wt^ homodimers (Figure 10.A). Co-transfections of endoglin with integrin-alpha-6 served as a negative control. The weakest interaction was found for ALK1, with only 12% of all cells analysed for FRET being clearly positive, with a relatively low mean FI of 166 and with a narrow range of value distribution of 61. Only 8% of the cells were in group 2. This indicates that this interaction is a rare event in non-stimulated CHO cells. In comparison to ALK1, ALK5 showed a more frequent and stronger protein interaction with endoglin^wt^. 36% of all cells were clearly positive, had a higher FRET index (mean FI 243), and a greater range of value distribution (260). 16% of the cells were in group 2. The strongest interaction of the three TGF-β receptors with endoglin^wt^ was found for TβRII. In this case, 62% of the cells were strongly positive (mean FI 293) with the largest range of value distribution of 330, and only 18% of the cells were in group 2. As expected, we found the strongest interaction for the endoglin^wt^ homodimer. In this case, 100% of the cells were strongly positive (mean FI 373) with a narrower value distribution range of 207, which was well separated from group 2.

Next, we tested the interaction of the membrane localized endoglin mutant S480C with the receptors (Figure 10.B). As a result of this, we observed an overall reduced interaction capability with this endoglin mutant and the TGF-β receptors, but the same relative trend was maintained compared to endoglin^wt^. The already low interaction between endoglin^wt^ and ALK1 was lost for endoglin^S480C^ and ALK1. Only 13% of the cells were in the FRET index group 2 (false or weakly positive) and no cells were found to be definitely positive. The Interaction with ALK5 was strongly reduced (mean FI 129). Only 13% of the cells were found to be positive, but with a very narrow index distribution range of 14, and 30% of the cells were in group 2. The strongest interaction of endoglin^S480C^ with the receptors was seen with TβRII, with a mean FI of 147 and with an index distribution range of 95. However, this seemed to be a very rare event, as only 3% of the cells were clearly positive, while 10% of the cells were in group 2. The strongest and most frequent protein interaction was seen for endoglin^wt^ and S480C, although it was not as strong as for the endoglin^wt^ homodimer. In this case, 65% of the cells were clearly positive with a mean FI of 239, and an index distribution range of 182, comparable to the endoglin^wt^ homodimers. 19% of the cells were in group 2.

Finally, we analysed the protein interactions of the TGF-β receptors with endoglin mutant G52V in the rER (Figure 10.C). As illustrated, there was no measurable interceptive effect of the rER residing mutant protein towards these receptors (none of the cells was clearly positive). The only receptor that generated FIs that were present in group 2 was TβRII with 8% of the cells. This was in contrast to the endoglin wt/mutant heterodimers that form in the rER. 51% of the cells were clearly positive (mean FI 222) with an index distribution range of 240, and 27% were in group 2. For the G52V homodimer, at least 75% of the cells were clearly positive (mean FI 204), covering a comparable index range as the heterodimers (216), and 15% were in group 2 (total 90%). The control receptors DRD1 and integrin-alpha-6 also appeared not to be retained in the rER by the endoglin mutant protein. Only 11% of the DRD1 expressing cells were in FI group 2 and 7% for IA6.

Taken together, the results demonstrate that in none of the tested combinations is the rER retained mutant-endoglin G52V capable of interfering with the analysed TGF-β receptors in the rER through heteromeric protein interaction, while the membrane present S480C mutant protein retains some interaction capability, but only with ALK5 and TβRII.

#### Image presentation of the FRET analyses

**Figure 11:**
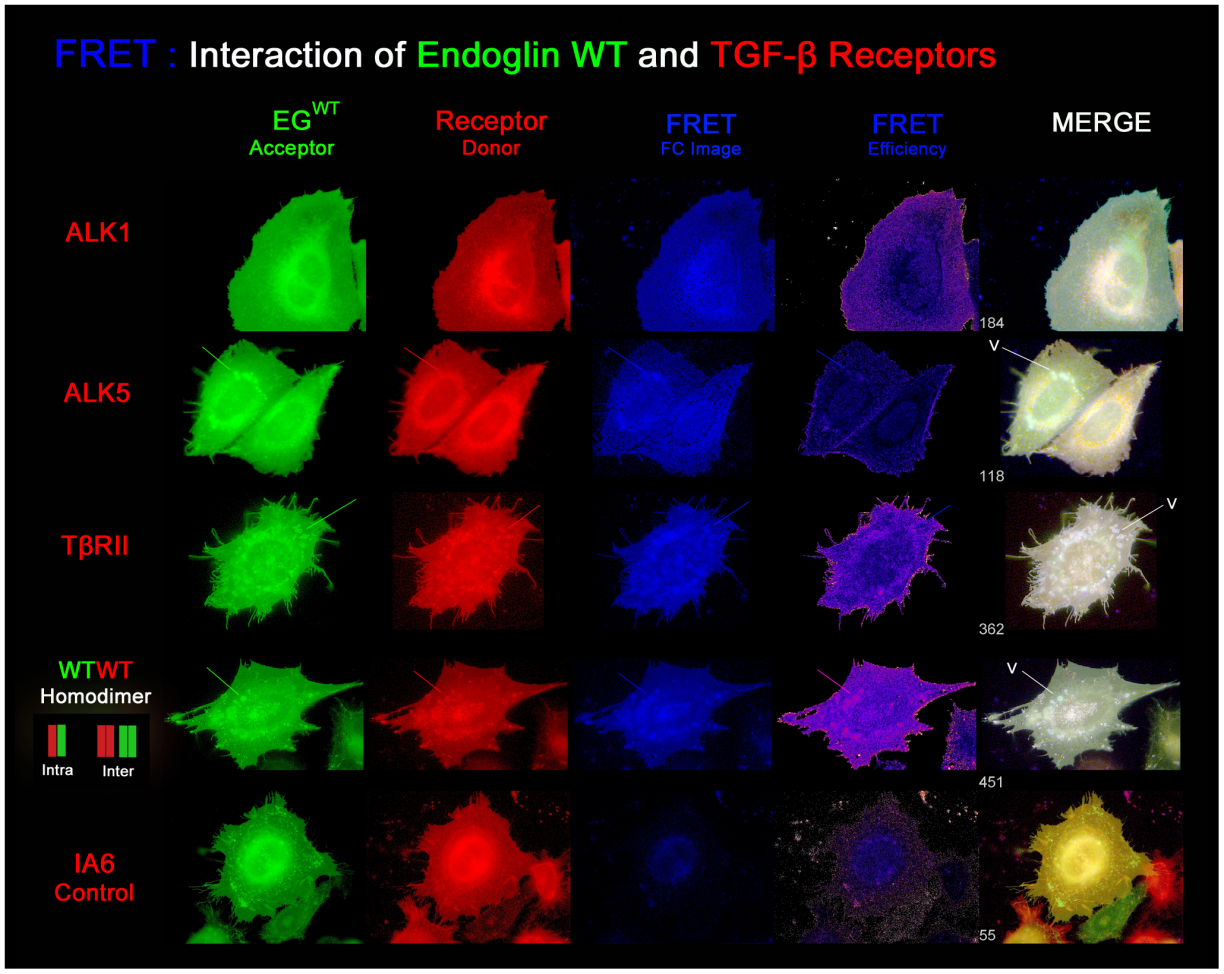
Endoglin^wt^ interacts to different degrees with the TGF-β receptors in the plasma membrane. Exemplary images of the FRET analysis showing interaction of endoglin^wt^-EYFP with the TGF-β receptors (ECFP-tagged, false coloured in red). The filter-corrected FRET image (FC) is shown in blue. The calculated FRET efficiency image is shown in an intensity-correlated manner (cold-warm). The 3-channel RGB-merge image becomes white in FRET-positive regions. Interaction is strongest between endoglin^wt^ and TβRII, followed by ALK5 and ALK1. Integrin alpha-6 (ECFP-tagged) does not interact with endoglin^wt^. In this case the merged image appears yellow. Endoglin homodimers show the strongest interaction due to inter- and intramolecular FRET in vesicles (V). For the other receptors, efficiencies in vesicles are not higher than in the cell membrane, as indicated by lines.

**Figure 12:**
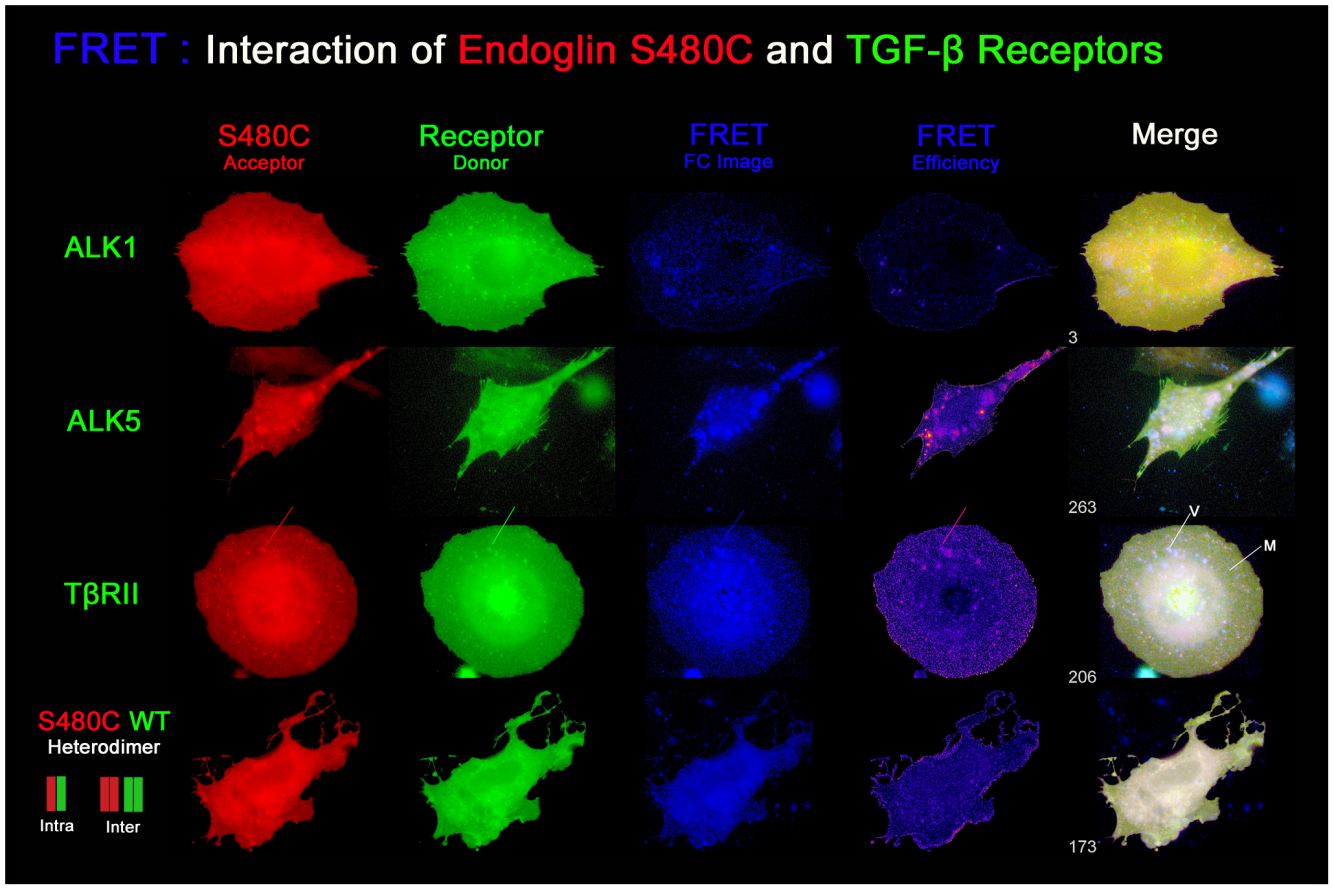
Mutant S480C shows impeded ability to interact with TGF-β receptors in the membrane. Interaction of mutant endoglin^S480C^ (FRET acceptor, false coloured in red) with the TGFB receptors (FRET donor, false coloured in green) is strongly reduced, although the relative trend is the same as seen before for endoglin^wt^. The strongest interaction was measured between S480C and TβRII, followed by ALK5. FRET signals can be observed in vesicles (V) and for the cell membrane (M). Interaction of endoglin^S480C^ with ALK1 is lost, there is no positive FRET. Unspecific FRET occurrence is shown by the blue colour in the merged image. Co-expression of endoglin^S480C^ and endoglin^wt^ produces the highest FRET signals due to intra- and intermolecular FRET within heterodimers and between homodimers.

**Figure 13:**
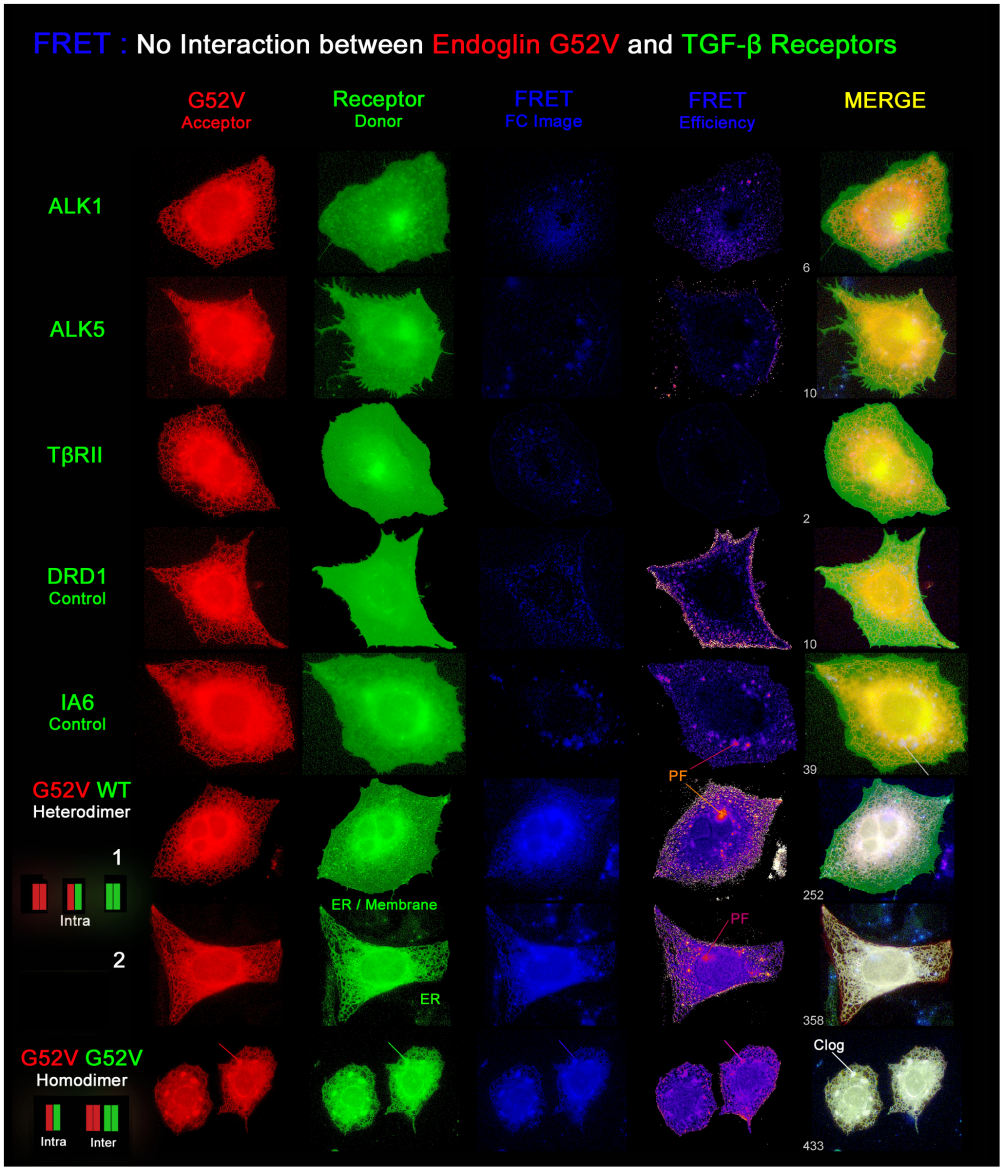
Mutant G52V does not interfere with TGF-β receptor processing in the rER. Co-expression of EYFP-tagged endoglin^G52V^ (FRET acceptor, false coloured in red) with the ECFP-tagged TGF-β receptors ALK1, ALK5, TβRII, or the negative controls DRD1 and IA6 (FRET donors, false coloured in green) did not yield positive FRET signals, in contrast to the co-expression with ECFP-tagged endoglin^wt^ and endoglin^G52V^. Despite the yellow colour in the merged image, the rER structure is not found in the receptor images. In the case of the negative controls, some rER residing IA6 or DRD1 proteins can be seen in the green channel, but the measured FRET efficiencies are below the threshold level considered to be positive. Signals from the filter-corrected FRET-FC image are unspecific pseudo-FRET signals (PF). These regions stay blue in the MERGE image and cannot be correlated to the acceptor image. Endoglin^G52V/wt^ heterodimer images: 1. Endoglin^G52V/wt^ heterodimers are visible in the rER while endoglin^wt/wt^ homodimers are present in the membrane. 2. In the case of excess mutant protein expression, no endoglin^wt^ homodimers are found in the membrane due to complete heterodimerization in the rER. Last line, endoglin^G52V^ homodimers: here mutant protein expression leads to rER clogging, shown by partially puffed up rER structures (clog).

Next to the diagrammatical presentation of the measurements, we present exemplary high resolution images of the FRET measurements (Figure 11–13). For each tested receptor combination, images of each channel, EYFP, ECFP, FRET, and the calculated FRET efficiency image are shown. Irrespective of the natural colours of the fluorophores, endoglin^wt^ is shown in green and the mutants are shown in red, whereas the TGF-β receptors are shown either in green or in red, depending on the tested combination. The filter corrected (Fc) FRET image is shown in blue. Besides the efficiency image (intensity correlated, cold-warm), all channels are auto-adjusted for display. Additionally, a 3-channel merged image is presented which results from RGB (red, green, blue) superimposition where the FRET index is indicated. In this image, receptor colocalization (yellow) and occurrence of FRET (white) are displayed at the same time and pseudo-FRET appears in blue ( = FRET> background & Donor/Acceptor absent).

The strongest interaction of endoglin^wt^ with the TGF-β receptors was measured with TβRII, followed by ALK5 and ALK1 (Figure 11), whereas no FRET was seen with integrin alpha-6 and endoglin^wt^. As can be expected, endoglin^wt^ homodimers produced the strongest FRET signals. In this case FRET occurs intramolecularly as well as intermolecularly. In the first case, a heterochromic endoglin homodimer is composed of one EYFP-carrying monomer and one ECFP-carrying monomer which produces intramolecular FRET. In the latter, FRET occurs between two homochromic homodimers that each carry either two EYFP or two ECFP fluorophores. This is promoted in compartments where such endoglin homodimers become compressed, due to spatial limitation such as transport vesicles (V). Because the population of homochromic and heterochromic homodimers is mixed, the two FRET effects act synergistically. In the plasma membrane, homochromic homodimers float dispersively or can become otherwise separated, lowering the occurrence of intermolecular FRET. For all three TGF-β receptors heteromeric FRET can be observed in the membrane and in transport vesicles, probably membrane directed. Here, FRET efficiencies in vesicles are mostly no higher than elsewhere in the cell, while in cells expressing endoglin homodimers the highest efficiencies are observed in vesicles, due to overlapping FRET effects.

The outcome of the FRET measurements between endoglin mutant S480C and the TGF-β receptors is shown in figure 12. Compared to endoglin^wt^, TGF-β receptor interactions are strongly reduced for mutant S480C, although the relative trend seems to be maintained. Again, the ability to interact is strongest between endoglin^S480C^ and TβRII if it occurs, followed by ALK5, but is completely lost for ALK1. Dimerization of S480C with endoglin^wt^ produces the strongest FRET signals, due to intra- and intermolecular FRET as explained above.

FRET measurements for possible interactions between mutant G52V and the TGF-β receptors are shown in figure 13. As discussed above, no protein interaction was found for any of the tested receptors. In contrast, endoglin homo- and heterodimers produced strong FRET. In the G52V/wt situation, intermolecular FRET becomes almost excluded as the green monochromic wild type homodimers are transported to the membrane, while the red monochromic G52V homodimers stay in the ER, resulting in a spatial sort out separation. As seen in lane 1 (green image), the heterodimers reside in the rER (G52V homodimers are red), where intramolecular FRET is produced (compare to FC image), and the green homodimers are in the membrane. In the case of excess mutant protein expression, the amount becomes sufficient to completely trap endoglin wild-type monomers in the rER through heterodimerization to such an extent that no more wild-type homodimers are formed to be present in the plasma membrane (Lane 2, green image); this cell is from the same sample as in lane 1. In the case of G52V-homodimers, again both FRET effects take place, as all dimer classes (homo- and heterochromic) are entrapped in the ER. In some cases, a high amount of protein leads to blown-up rER structures due to rER clogging; more protein is synthesized than can be degraded and removed from the ER through possible ERAD mechanisms (ER associated protein decay, see discussion).

## Discussion

In this study, we have investigated the expression and cellular localisation of HHT-1 causing endoglin missense mutant proteins. Furthermore, we have tried to identify the molecular mechanism of how these mutants might interfere with normal endoglin function. By employing different fluorescence microscopy techniques, we have shown that all of the mutant proteins carrying an EYFP tag investigated here were equally present when transiently or stably expressed in CHO cells or in HMEC-1 or REC endothelial cells. Most of these mutant proteins were intracellularly trapped within the rER without observable membrane localisation. This was demonstrated by colocalisation experiments with the rER localised quality control chaperone calnexin. However, two of the mutant endoglin proteins investigated, S480C and R571H, localised in the plasma membrane like the wild-type protein. The observation of endoglin missense mutant protein localisation in the rER is in accordance with other research [52] in which a large number of endoglin missense mutations was also analysed by fluorescence microscopy. In that study, however, the mutant proteins W149C and G413V, expressed in HeLa and HEK293 cells, were found to predominantly localise in the membrane. This is different to our observation, where these proteins localise in the rER when expressed in CHO, HMEC-1 or REC endothelial cells. In some cases, however, mutant G413V sometimes showed slight membrane localisation.

We have performed control experiments to ensure that a C-terminal tag to endoglin by itself might not already interfere with the processing of a membrane protein. Whether or not basal phosphorylation of endoglin's intracellular domain might also play a role in this process and if a tag might block that basal phosphorylation we do not know and was not investigated in this study. However, we have shown that the EYFP-tagged wild-type endoglin is regularly integrated in the cell membrane suggesting that such a tag has in general no major negative effect on protein processing and trafficking from the rER to the cell membrane. In addition, a previous study had already shown that phosphorylation does not play a direct role in the expression or maturation of endoglin [57].

Interestingly, our attempt failed to directly detect transiently transfected untagged missense mutants by immunostaining with the endoglin specific monoclonal antibody SN6, which prompted us to use EYFP-tagged proteins for microscopical immuno-detection. This analysis clearly showed that most of the mutant proteins that are only present in the rER, as visualized using the EYFP tag, failed to react with this antibody, whereas the membrane localised mutants S480C, R571H, the wild-type endoglin protein, and the rER localised G413V mutant were readily detectable. In addition, our analysis indirectly delivers information about the conformation of the missense mutant proteins. It suggests that many missense mutations lead to misfolding of the proteins in such a way that they become detected by the protein quality control machinery (e.g. Calnexin). As a result, they are trapped in the rER, and probably subjected to the ER associated protein decay (ERAD), as also proposed by others previously [52]. The inability to react with SN6 suggests that the misfolding leads to the loss of the antibody binding-epitope. This might explain why, in previous studies, several missense proteins were found to be absent in patient cells or when transiently expressed. In these studies, two monoclonal antibodies, P3D1 and P4A4, were predominantly used for immunostaining, co-IP and western blot. Antibody P4A4 recognizes the same epitope (AS Y277-G331) [53] within the extracellular region of the wild-type protein as the SN6 antibody we used (AS Y277-P338) [48].

Based on biochemical studies, it had already been suggested [40] that endoglin missense mutants dimerise with wild-type endoglin. By using BiFC, in order to analyse endoglin/endoglin mutant dimerisation, we could now definitely prove that all missense proteins investigated here form homodimers with themselves and heterodimers with endoglin^wt^. Therefore, all three different endoglin dimers are likely to be present at the same time: mutant homodimers, wild-type homodimers, and mutant/wild-type heterodimers. To what extent this might contribute to a stronger reduction of membrane endoglin^wt^ (theoretically 67%) than to be expected by haploinsufficiency (50%) in patient samples remains to be carefully investigated.

The wild-type/mutant heterodimers, as visualized through BiFC in CHO cells, showed that they behaved the same way as the respective mutant homodimers in terms of localisation and antibody reactivity. This suggests a dominant influence of the mutant monomer over the wild-type endoglin protein, causing in many cases an entrapment in the rER by the ER quality control. Heterodimerisation in the rER proved to have a strong interfering effect towards the endogenous endoglin^wt^ monomer molecules. When we transiently or stably expressed rER-trapped missense mutants in HMEC-1 cells, we observed a reduction of about 40–50% of the endogenous endoglin^wt^ homodimers on the cell surface, while expression of endoglin^wt^ or endoglin^Δcyto^ led to a total increase of about 180%. Interestingly, however, the expression of the membrane localised mutant S480C showed only a slight increase of about 20% of total stainable surface endoglin, suggesting that high S480C protein levels were not well tolerated by the cells during the stabilization process for this protein. Indeed, it was extraordinarily difficult to generate a stably expressing S480C HMEC-1 cell line. All other endoglin variant expressing HMEC-1 cell lines were generated during a time span of approximately 3–4 weeks, whereas it took more than 8 weeks and repeated transfections to generate a stable S480C cell line. In addition, once stable transfected cell lines were established, all mutant cell lines showed comparable amounts of expressed mutant proteins, but only 50–60% of that of the ectopic wild-type level. This observation might further support the idea that only a certain amount of mutant endoglin protein is tolerated and that increased expression of mutants leads to cell death.

The reduction of surface endoglin through rER heterodimerisation also had a strong impact on endoglin TGF-β signal modulation towards the ALK5-SMAD-3 pathway, as measured by the CAGA luciferase reporter assay using non-tagged endoglin constructs. When we transiently expressed wild-type endoglin in HMEC-1 or in REC rat endothelial cells, the natural ALK5 mediated reporter signal became almost deleted, which is in accordance with another study [54]. When expressing the rER-trapped G52V mutant, the signal was not changed or even raised over the natural non-endoglin inhibited ALK5 signal. This suggests that, depending on transfection efficiency of the mutant construct, the amount of membrane wild-type endoglin is sufficiently reduced to promote a TGF-β induced ALK5 signal. When the reduced amount of cell surface wild-type endoglin is still beneath a sufficient threshold, however, the reporter signal stays unchanged. The expression of the membrane localised mutant S480C led to a signal decrease by only about half of the natural ALK5 mediated reporter activity. Based on this result and our FRET data, and the fact that three types of dimers (wt/wt, wt/S480C, S480C/S480C) are present on the cell surface, we hypothesize that the mutant/wild-type heterodimer still possesses some remaining inhibitory activity, whereas the S480C homodimer suffers a complete loss of function. Otherwise, the same result should have been generated as with the rER-trapped mutant G52V. In this case, the same amount of functional wild-type homodimers (theoretically 1/3) only would have remained active in the membrane. In the case of full functionality of the mutant protein, the same signal decrease as with wild-type endoglin should have been generated. Surprisingly, when we analysed the endoglin^ΔC^ variant, we observed a similar signal decrease as we did with endoglin^wt^, although the signal remained slightly higher. This suggests that it is especially the extracellular part of endoglin which is responsible for signal modulation, which had also been proposed in a previous study [27].

We also investigated the influence of endoglin^wt^ and endoglin mutant proteins G52V and S480C on cell proliferation in stabilized L6E9 rat myoblasts and stabilized CHO cells. Endoglin has been reported to enhance cell proliferation by abrogating the inhibitory/antiproliferative effect of TGF-β-1 in different cell types [49], [50]. In our experiments, in the absence of TGF-β1, stable endoglin^wt^ over-expression showed, in both cell types, a general pro-proliferative effect even under low serum conditions. In stabilized CHO cells, endoglin mutants S480C and G52V also increased cell proliferation, although S480C less so than endoglin^wt^ and G52V even less than S480C. Surprisingly, the endoglin variant endoglin^ΔC^ without the endoglin cytoplasmic domain mediated an even stronger proliferation than endoglin^wt^. This would suggest a predominant function of the extracellular domain concerning proliferation, whereas the intracellular part might play a role in the intracellular regulation of functions of the endoglin molecule that leads to a less strong proliferative effect.

A various number of proteins have been reported to play a role in cell migration and adhesion that interact with the intracellular part of endoglin, like Zyxin [15], ZRP1 [16], TcTex2β [17], GIPC [26], or, probably most important in this case, β-Arrestin2 [55], which has also been brought into context with the pro-proliferative c-myc gene [56]. It was reported that association with β-Arrestin2 leads to an internalization of the endoglin/β-Arrestin2 complex in endocytotic vesicles and mediates inhibition of cell migration. Next to direct protein interaction within the cytosolic domain of endoglin, different sites of phosphorylation have been reported for the cytosolic part [57], which has also been related to the inhibition of endothelial cell migration owing to endoglin phosphorylation by ALK5 [58]. In all these reports, it is the cytosolic domain of endoglin that was needed for the inhibition of cellular migration, exactly what is lacking in the endoglin^ΔC^ construct. Cell migration and cell proliferation are two different cellular activities, but they do also have processes in common that might be influenced by endoglin, e.g. detachment from the extracellular matrix and attachment afterwards, or composition of focal adhesions. It is possible that the pro-proliferative effect of endoglin is regulated by its cytosolic domain, depending on or mediated by various proteins and phosphorylation of specific amino acids in which receptor internalization may also play an important role. Thus, if the intracellular part, which is responsible for fine-tuning, is missing, then this might lead to the observed enhanced proliferation. The endoglin mutants S480C and G52V also enhanced proliferation in CHO cells. This points towards some remaining functionality of the S480C mutant homodimer protein regarding proliferation, whereas, when looking at its inhibitory activity on ALK5 signalling, the functionality of the mutant homodimer appears to be impeded, as explained above. The weak pro-proliferative effect of the G52V mutant seems hard to explain in view of the most likely role of the endoglin extracellular domain in this process. This protein remains inside the rER. Thus, proliferation enhancement cannot solely be addressed to the extracellular domain. Right now, we can only speculate that the weak pro-proliferative effect of G52V is mediated through the intact cytosolic domain that is protruding into the cytoplasm and still able to bind regulatory proteins involved in cell proliferation. ER localised proteins have been reported to interact with plasma membrane localised proteins and to establish direct connections between these two compartments [59]. Therefore, the interaction of the cytosolic tail of, for example, mutant G52V with proteins associated with, or integrated into the plasma membrane cannot be excluded. Our findings suggest that proliferation is mediated through the extracellular part of endoglin, which might be further finely regulated by its intracellular domain.

Interaction in the plasma membrane between endoglin^wt^ and the three TGF-β receptors ALK1, ALK5, or TβRII has been reported previously [25], [40], [60], based on biochemical co-IP experiments. Therefore, in view of the interfering effect of rER trapped endoglin missense mutant proteins towards the wild type protein, it seemed useful to analyse whether this would also apply to other TGF-β transmembrane receptors during their synthesis and processing in the rER in the presence of co-translated endoglin missense mutant proteins. Furthermore, whether endoglin reaches the cell surface as a monomer or a homodimer, already preformed in the rER and Golgi and whether endoglin/TGF-β receptor oligomers are already assembled in the rER during protein synthesis, is not well established. In order to test this, we used FRET for the measurement of protein interactions in a non-endothelial system (CHO cells). CHO cells were used in order to exclude interfering effects of endogenously expressed endoglin, as, for example, in endothelial cells.

The FRET analyses show that endoglin dimers are formed in the rER and are transported as dimers to the membrane, as demonstrated by the strong FRET signals seen in transport vesicles. As expected, we were also able to measure TGF-β receptor interactions with endoglin^wt^, but to different degrees. The strongest interaction was with the type II receptor TβRII, while interactions with the type I receptors ALK5 and ALK1 were less strong. Vesicle-related FRET was also measured for endoglin^wt^/TβRII, as well as for endoglin^wt^/ALK5, but not for endoglin^wt^/ALK1 receptor pairs.

In the non-endothelial system used here, spontaneous interaction with ALK1 seems to be a rare event and the interaction is only weak. This points towards a usually low affinity of the two proteins for each other, suggesting that endoglin and ALK1 interactions are most likely ligand dependent. However, we cannot exclude that this might be different in endothelial cells or that the EYFP- and ECFP-tags cause structural changes, preventing efficient endoglin and ALK1 interaction and therefore also FRET. Ligand-independent interaction with ALK5 seems to be more favourable, as it has been observed more frequently and displays higher FRET efficiencies.

More important are the results of our analysis of whether the missense mutants affect trafficking of other rER processed proteins or not. We tested the endoglin mutant G52V, as one representative of rER retained missense mutants, and mutant S480C for membrane localisation. Again, dimerisation was confirmed for endoglin wild-type/mutant heterodimers, as well as mutant homodimers with varying efficiencies. Mutant homodimers and mutant/wild-type heterodimers displayed lower efficiencies than wild-type homodimers. When investigating the membrane localised endoglin mutant S480C, we observed the same trend for interaction with the TGF-β receptors as for endoglin^wt^. The already low interaction with ALK1 became completely lost, while there was still some measurable interaction with ALK5 and TβRII. However, to our surprise, in the case of the rER trapped mutant G52V the reduced or lost receptor interaction was even more dramatic, no interaction with the TGF-β receptors could be measured any more. Next to the loss of specific protein interactions, we also could not observe any general negative effect on protein synthesis that might result from rER clogging due to the mutant proteins. The other two rER processed transmembrane proteins DRD1 and IA6, which do not interact with endoglin^wt^ in the membrane, were also not retained in the rER by the mutant endoglin, as demonstrated by the absence of specific FRET signals. Our results suggest that in HHT1 at first glance only the function of endoglin is affected, and that the majority of the missense mutants do not interfere with other TGF-β receptors. Thus, our findings are in complete agreement with the signalling imbalance model [61], [62] of tilting the ALK1 and ALK5 signalling balance towards a non-endoglin modulated TGF-β1 activated ALK5/TβRII pathway.

In summary, our results show that the endoglin missense mutant proteins are capable of homodimerisation as well as heterodimerisation with endoglin^wt^. In consequence, for those mutants that are expressed at the cell surface this leads most likely to reduced or abrogated wild-type endoglin functionality. In the case of the missense mutants that are trapped in the rER, this leads to a reduced amount of intact wild-type endoglin homodimers at the cell surface, since these mutants act in a dominant negative fashion upon the wild-type endoglin protein. So far, since this was not part of our analysis, we do not know whether the rER retained mutant homodimer and mutant/wild-type heterodimer protein complexes are degraded by the ERAD system, although this seems to be most likely. There are numerous examples in the literature of protein degradation of misfolded proteins by the ERAD machinery. Thus, it would be interesting to investigate to what extent missense mutant proteins accumulate in the rER and might induce ER stress and promote ERAD or even cell death during angiogenesis or angiogenesis-like processes, when endoglin expression is highly up-regulated. In agreement with this, after 48 hours we repeatedly observed a highly reduced number of cells expressing the G52V mutant compared to earlier time points and compared to cells still expressing endoglin^wt^ or ALK1, ALK5 or TβRII in the same cell culture dish (data not shown). This suggests that mutant endoglin expressing cells die earlier, perhaps due to ER stress.

Disturbance or reduction of normal endoglin function seems to be sufficient for HHT1 phenotypes, consequently also leading to a reduced amount of full functional endoglin^wt^ homodimers in the membrane. However, of all known HHT1 mutations that affect the endoglin gene, missense mutations represent only a proportion of about 20%. In this context, it becomes particularly interesting to look at the ALK1 gene responsible for HHT2. Here, about 50% of the mutations are missense mutations, which affect the extra- as well as intracellular part of the ALK1 protein. A recent study found that many ALK1 missense mutants are also retained in the ER [63]. In this context it would be extraordinarily interesting to investigate if effects similar to those observed in our study for endoglin apply to ALK1, in terms of ER interference due to heterodimerisation leading to a membrane reduction of ALK1 wild-type protein dimers.

As a last thought, the endoplasmic reticulum (ER) is the major site in the cell for protein folding, maturation, quality control, and trafficking, and a failure of such owing to the accumulation of unfolded or misfolded proteins leads to ER-stress inducing the unfolded protein response (UPR) or ERAD. There is emerging evidence that chronic ER-stress intersects with many different inflammatory and stress signalling pathways (reviewed in [64]). ER stress responses and their intersection with inflammatory pathways might be important in the context of metabolic homeostasis and disease and might provide a further explanation for the variety of disease phenotypes and penetrance among HHT patients, depending on the type of mutation.

## Material and Methods

### Cell culture

The following cell lines were used in this study, CHO-K1, HMEC-1 [65], L6E9 and RFPEC.

CHO-K1 were obtained from DSMZ GMBH,Germany (DSMZ-No. ACC-110). L6E9 rat myoblast cells [66], [67] were a kind gift by Dr.Carmelo Bernabeu (Centro de Investigaciones Biológicas, CSIC, Madrid). Rat fat pad endothelial cells, RFPEC (REC) [68], [69] were a kind gift from Prof. T. Wieland (Institute of Pharmacology and Toxicology, Medical Faculty Mannheim, University of Heidelberg, Germany). Cells were cultivated in an incubator under 5% CO2 atmosphere and at 37°C using the appropriate media, supplemented with 10% FCS if not otherwise specified. CHO-K1 were grown in F12 medium. REC, HMEC-1 and L6E9 were grown in DMEM. All media and serum was purchased from PAA, Austria.

### DNA Constructs

#### EYFP- and ECFP-tagged receptor constructs

References of all endoglin missense mutant sequences described here can be accessed on the online HHT Mutation Database (http://www.arup.utah.edu/database/hht/), apart from mutations S480C and R571H, identified by Dr.Jonathan Berg (Dundee, UK; personal communication). The amino acid change S480C was identified in a Scottish HHT family and R571H was identified in a patient from Morocco with a single sporadic brain AVM.

Wild-type and mutant endoglin (G52V, W149C, A160N, L306P, G413V) constructs in the pCMV5 vector and the endoglin-Δcyto (ΔC) construct have been described previously [41]. Further mutations and variations, G191D, S480C and R571H, were generated by site-directed mutagenesis using the pCMV5-endoglin wild-type construct as a template. In order to generate endoglin fluorescence-tagged fusion proteins, coding sequences were transferred from the pCMV5 vector constructs into the EcoR1/BamH1 cloning sites of the pEYFP-N1 and pECFP-N1 vectors (Clontech). All constructs were verified by sequencing. The fluorescence-tagged TGF-β receptors, ALK1-EYFP, ALK5-EYFP and TβRII-EYFP have been described previously [70].

The fluorescence-tagged dopamine receptor D1 (DRD1-EYFP) was a kind gift of Dr. A.Holloshi (IMZ, Mannheim University of Applied Sciences, Germany). The Integrin-alpha-6-ECFP construct was a kind gift of Dr. A.Sonnenberg (Netherlands Cancer Institute).

#### Endoglin BiFC constructs

BiFC vectors were generated [47] by removing the original EYFP sequence from the vector (EYFP-N1) using the restriction sites Age I (5′) and Not I (3′). Subsequently, BiFC fragments coding for the EYFP amino acids 1–172, and 173–238 were cloned into Age I/Not I site of the modified EYFP-N1 vector.

BiFC EYFP fragments were generated by PCR using the original vector pEYFP-N1 as a template and the primer combinations listed below to generate the BiFC vectors named BiFC-N172 and BiFC-173C: **Fragment N172** (aa 1–172): primer pair EYFP-FWD+Age1 5′-CACCGGTCGCCACCATGGTGAG-3′/EYFP-aa172 REV+Not1 5′-CGCGGCCGCCTCGATGTTGTGGCGGATCTTG-3′; **Fragment 173C** (aa 173–238): primer pair EYFP-aa173 FWD+Age1 5′-CACCGGTCATGGACGGCAGCGTGCAGCTCG-3′/EYFP-REV+Not1 5′-CGCGGCCGCTCTTGTACAGCTCGTCCATGC-3′. Wild-type endoglin and mutant sequences were transferred from the above-mentioned endoglin-pEYFP-N1 constructs into the EcoR1/BamH1 restriction sites of the BiFC vectors. Finally, constructs were verified by sequencing.

### Cell line transfection and generation of stable cell lines

CHO cells were transfected using the JetPei Reagent (Polyplus); for other cell types FUGENE-HD (Roche) was used. All transfections were conducted according to the respective manufacturer's instructions. In order to generate stable transfected CHO-K1 and HMEC-1 cell lines, cells were transfected at a confluence of 75% in 6-well plates. After 48 h of expression, cells were selected and expanded under 800 µg/ml G418-sulfate (Sigma) in T75 flasks. Stabilized cells were sorted for green fluorescence by FACS with a BD FACS Aria III cell sorter, and subsequently kept under 400 µg/ml G418-sulfate for further culture expansion. During experiments G418 was omitted.

### Immunofluorescence and live cell imaging

For microscopy of stable transfected cells with EYFP-tagged receptor constructs, cells were seeded on glass cover slips to reach maximally 50% confluence the next day. For microscopy of transient transfected cells, cells were allowed to attach and spread on the glass for at least 4 h. After cell attachment and spreading, cells were washed in PBS to remove debris and unattached cells before transfection. Cells were allowed to express constructs for 24 h. Cells were washed in PBS and fixed in 4% PFA for 10 min. The PFA was removed and the cells were washed 2x in PBS; residual PFA was washed out two times for 5 min with a 50 mM Tris-Cl (pH 7.5), 100 mM NaCl quencher solution. When necessary, membranes were permeabilized using 0,2% Triton X-100 in PBS for 10 minutes' shaking. Cells were blocked for 30 minutes in PBS/3% BSA and washed 1x in PBS. Primary antibody was diluted in PBS/1%BSA and allowed to bind for 60 min under gentle shaking.

For indirect endoglin staining, the mouse monoclonoal antibody SN6 from Acris (SM1177P) was used in a working dilution of 1∶500. For calnexin staining, a monoclonal antibody from mouse (Abcam ab31290) was used in a dilution of 1∶500. After primary antibody incubation, cells were washed three times in PBS for 5 min. Secondary antibody (Goat anti Mouse, TRITC, Sigma T-7657) was used in a dilution of 1∶200 in PBS/1% BSA and allowed to bind for 60 min. After secondary antibody incubation, cells were washed in PBS 5 times for 5 min under gentle shaking. Cover slips were mounted on glass slides with Mowiol/Dabco (Sigma).

For live cell imaging, CHO cells were seeded on glass dishes one day before transfection. Cells were transfected with 50 ng for each EYFP- and ECFP-tagged construct per 100 000 cells and kept in medium containing 10% FCS. For live measurements, F12 medium was replaced by Leibowitz medium (PAA) 24 h after transfection.

### Cell proliferation assays

Cell proliferation assays with stabilized L6E9 and CHO-K1 cell lines were conducted in 96-well plates at different FCS concentrations (1%, 2% and 5%). Growth rates were monitored over a period of 96 hours, with recording points at 0 h, 24 h, 48 h, 72 h, and 96 h. Prior to the assays, cells were seeded at 50% confluence and kept for 24 hours in medium containing only 5% FCS. Cells were trypsinized and trypsinization was stopped by harvesting the cells with 10% FCS containing medium. To ensure that exactly the same cell numbers of the different stable cell lines were seeded, cells were counted in duplicates with a Vicell-counter (Beckman-Coulter).

For each assay, 5 000 cells in 200 µl medium with the indicated FCS concentration were seeded per well in 96well plates. For each time point and FCS concentration, one plate was prepared, containing every cell line. Cells were seeded in the maximum possible number of wells available. When testing the CHO cell lines, each cell line was seeded in 10 wells. When testing the L6E9 cell lines, each cell line was seeded in 20 wells. The remaining outer wells were left free and filled with PBS as an evaporation barrier. After 4–6 h, the first plate was removed and fixed in 4%PFA for time point zero and equal cell seeding determination. Every 24 h, one plate was removed and then fixed. After collection of the time series, plates were washed in PBS and stained with DAPI for 5 min. Plates were washed 2x in PBS and measured, containing 100 µl PBS per well (Ascent Fluoroscan). Alternatively to the DAPI staining method, L6E9 growth rates were measured using crystal violet as stain. In this method, PFA fixed plates were washed in distilled water and subsequently dried. Plates were stained with 50 µl 2% crystal violet solution and incubated for 15 min. Stain was removed and plates were washed in distilled water two times and dried. Plates were filled with 100 µl/well of 10% acetate to dissolve the stain and extinction coefficients were measured in a plate reader. Proliferation profiles were normalized for time point zero and compared to the proliferation of control (mock) cells.

### Luciferase-based promoter reporter assay

Native HMEC-1 and REC cells were seeded in parallel to confluence under 5%FCS in 12-well plates and allowed to attach for at least 2 h. After cell attachment and spreading, cells were washed in PBS to remove debris and unattached cells. For co-transfection, 500 ng of the CAGA-reporter [71] and 500 ng of the respective non-tagged endoglin construct (in pCMV5 vector) each were used for 500 000 cells. Construct concentrations were equilibrated by gel electrophoresis and densitometric quantification (Lumi Imager). After 16–18 h, cell samples were trypsinized and seeded into 10wells (100 µl/well) each in a 96-well plate under 2% FCS. After 6 h, medium was exchanged and cells were stimulated with 4 ng/ml TGF-β1 (2% FCS). After a stimulation period of 18 h, cells were lysed in luciferase buffer according to the manufacturer's instructions (Steady Glow, Promega) and lysates were transferred and measured in white plastic 96-well plates (Lumi Imager). The assay was repeated 6 times.

### Quantification of BiFC by Flow-Cytometry

CHO-K1 cells were seeded in 24-Well plates to confluence (160.000 cells/well). Cells were cotransfected with 100 ng of each BiFC construct in all possible combinations as indicated. After an expression time of 24 h cells were trypsinized and counted by flow cytometry (Partec Flow Cytometer, Partec Germany). Triplicates of non-transfected cells in each experiment were gated for zero fluorescent counts and the autofluorescence limit was set. Percentage of fluorescence positive cells within a sample was determined. The experiment was repeated four times. At least 20.000 cells were counted per sample.

### Microscopic analysis/Colocalisation/FRET

Image acquisition was conducted with a Zeiss Axiovert 200 Fluorescence microscope (Axiovision software) and an Axiocam camera. In general, microscopical images were taken with a 63x magnification lens for resolution of subcellular localisations. Quantitative measurements were conducted under constant exposure times. Images were background corrected. Measurements of membrane endoglin in stabilized HMEC-1 were conducted globally using a 20x lens of a 70% confluent cell layer. Image analysis was conducted using the ImageJ software.

### Colocalisation analysis

Microscopic images of living cells were taken 24 hours after transfection. Pearson correlation coefficients for colocalisation analysis were gathered using the “Manders Coefficients” plugin for ImageJ which is implemented in the MBF *ImageJ for Microscopy* Collection available from the official ImageJ website. The experiment was repeated three times. At least 30 images of each sample were analysed.

### FRET

100 000 CHO cells were co-transfected with (50 ng) donor and (50 ng) acceptor receptor fusion constructs. After 24 h, medium was removed and living cells were analysed by fluorescence microscopy in a HEPES buffered physiological ringer solution (NaCl: 140/KCl: 2/CaCl_2_ x 6H_2_O: 2/MgCl_2_ x 6H_2_O: 1/d-glucose: 20/HEPES: 10 [mM]). Microscopic images were background subtracted. The FRET image was filter corrected ( = FC Image). The efficiency image was calculated according to formulas reported jn [72], using a FRET/EYFP correlation factor (g) obtained through acceptor photobleaching calibration. For this, we used a cytosolic EYFP-28 amino acids-ECFP fusion protein transfected into CHO cells (kindly provided by Dr. A. Holloschi, our institute). For efficiency determination, we used the histogram mode value (value of highest abundance) of the calculated efficiency image instead of the mean value. This proved to be more representative, as a few high-value artefact pixels can have a strong influence on the mean value calculation. Image analysis and FRET image calculation were performed automatically using the ImageJ macro language. FRET results were obtained from several repeated individual (single sample) and combinatorial (multiple samples) transfection assays (at least three independent experiments) and at least 30 cells per sample were analysed. The most representative measurement of a given receptor combination is presented in the diagramme.

## Supporting Information

Figure S1 **Fluorescence-tagged endoglin^wt^ localises in the plasma membrane.** In order to test for the correct plasma membrane localisation of an EYFP-tagged endoglin wild type protein, CHO cells were transfected with an endoglin^wt^-EYFP expression construct. After an expression time of 24 hours non-permeabilised cells were fixed and immuno-stained with the endoglin-specific monoclonal antibody SN6 (TRITC-labelled) in order to detect only the membrane present endoglin protein. For comparison, endoglin^wt^-EYFP transfected CHO cells were permeabilized and stained with SN6, also showing intracellular structures like vesicles (line) related to endoglin's intracellelular localisation, in contrast to non-permeabilised cells demonstrating membrane surface staining only.
(TIF)

Figure S2 **Colocalisation of endoglin^wt^ and mutants visualized with interchanged fluorophores in CHO cells.** CHO cells were co-transfected with endoglin^wt^-EYFP and ECFP-tagged mutants. This variation enhances visibility of the rER retained endoglin^wt^ proportion through the plasma membrane but lowers visibility of the mutant proteins within the rER due to differences in fluorophore quantum yield (brightness).
(TIF)

Figure S3 **Quantitative colocalisation analysis of endoglin^wt^ & mutants.** Quantification of colocalisation between endoglin mutants and endoglin^wt^ was performed using Pearson correlation coefficients of two channel fluorescence images. CHO cells were co-transfected as indicated and live cell images were taken after 24 hours of expression. Samples are shown in two different groups depending on localisation of the respective endoglin mutant proteins either in the rER or in the plasma membrane. Within the groups no significant differences can be observed among the mutants. However, membrane localised mutants produce stronger colocalisation values (∼99) with endoglin^wt^ than ER localised mutant proteins (∼90). Co-transfection of the DRD1 receptor together with the ER trapped mutant G52V or together with endoglin^wt^ results in a coefficient of ∼70. **Av**: Group average. The results represent mean values of three experiments.
(TIF)

Figure S4 **Colocalisation of endoglin^wt^ and mutants in rat endothelial cells.** Rat endothelial cells (RECs) were cotransfected with endoglin^wt^ - ECFP and endoglin mutants (EYFP). The localisation of mutant proteins and endoglin wild-type in RECs is identical to the localisation as observed in CHO cells.
(TIF)

Figure S5 **Co-transfection of endoglin^wt^ and endoglin^G52V^ with the dopamine receptor D1 (DRD1).** CHO cells were co-transfected with endoglin^wt^-ECFP or endoglin^G52V^-ECFP together with the DRD1-EYFP expression construct. As displayed the DRD1 receptor is not retained in the rER by the G52V endoglin mutant protein. Furthermore DRD1 shows a different localisation pattern than the endoglin^wt^ protein. This leads in both cases to lower Pearson correlation values as shown in figure S3.
(TIF)

Figure S6 **Determination of BiFC specificity through endoglin dimerisation by Flow-Cytometry.** *Technical details* : In order to test the complementation specificity caused by endoglin dimerization against unspecific auto-complementation of the BiFC fragments, cells were co-transfected with the corresponding compatible BiFC partners as indicated and counted by flow cytometry after an expression time of 24 hours. The experiment was set up containing two different test variations. **Variation A**: The *N-terminal* BiFC fragment fused to the different endoglin variants was co-expressed with its C-terminal BiFC counterpart either alone [1] or fused to endoglin^wt^ [2] or fused to the DRD1 receptor [3]. **Variation B**: The C-*terminal* BiFC fragment fused to the different endoglin variants was co-expressed with the corresponding N-terminal BiFC fragment as in variation A [5], [6], [7]. **Endoglin homodimers** (green, [2], [4], [6]) can not be classified within these two variations as interchanging BiFC fragments does not apply. **Controls** [8]: (**F+F**) putative auto-complementation by BiFC fragments alone. (**D+C**) DRD1 + C-terminal BiFC fragment. (**D+N**) DRD1 + N-terminal BiFC fragment. (**D+D**) putative auto-complementation of DRD1 receptors (DRD1 is a monomeric receptor). (**nt**) non-transfected cells as a gating control. The average (**Av**) for each group is indicated (grey bar). The diagramme shows mean values of 4 independent experiments. *Result* : Occurrence of BiFC produced by endoglin dimerisation is significantly higher than produced by intrinsic fragment auto-complementation [1], [5] or when expressed together with the DRD1 receptor [3], [7]. The auto-complementation background [5] becomes diminished (∼0) when co-expressing the DRD1 receptor BiFC counterpart [7], instead of co-expressing the unfused BiFC fragments [5], which resembles the artificial character using single BiFC fragments as a control for unspecific fragment auto-complementation.
(TIF)
