# Supplementary figures and images for: Investigation of Endoglin Wild-Type and Missense Mutant Protein Heterodimerisation Using Fluorescence Microscopy Based IF, BiFC and FRET Analyses
Source: PLoS One. 2014 Jul 31;9(7):e102998. doi: 10.1371/journal.pone.0102998 (PMC4117486; doi:10.1371/journal.pone.0102998)

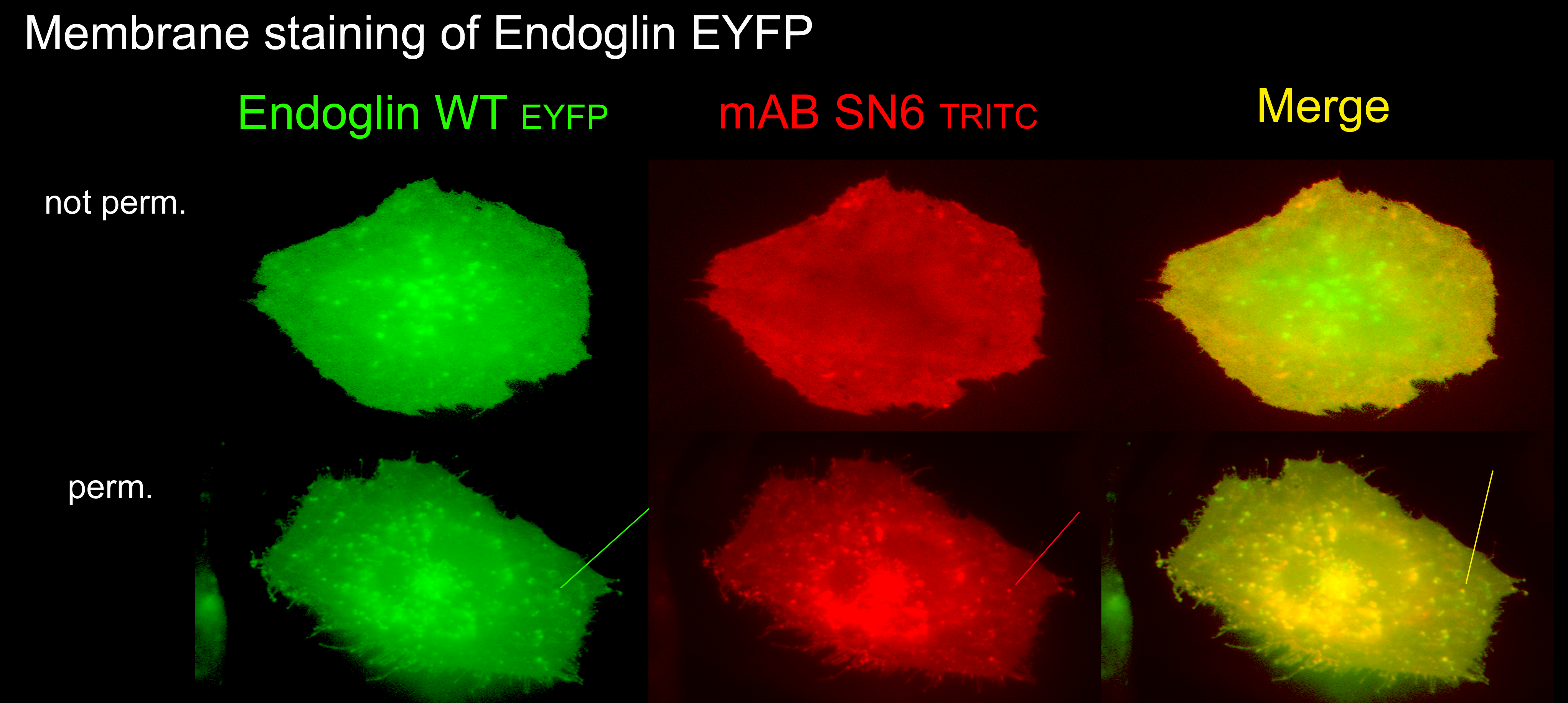

Supplement: Figure S1 — Fluorescence-tagged endoglinwt localises in the plasma membrane. In order to test for the correct plasma membrane localisation of an EYFP-tagged endoglin wild type protein, CHO cells were transfected with an endoglinwt-EYFP expression construct. After an expression time of 24 hours non-permeabilised cells were fixed and immuno-stained with the endoglin-specific monoclonal antibody SN6 (TRITC-labelled) in order to detect only the membrane present endoglin protein. For comparison, endoglinwt-EYFP transfected CHO cells were permeabilized and stained with SN6, also showing intracellular structures like vesicles (line) related to endoglin's intracellelular localisation, in contrast to non-permeabilised cells demonstrating membrane surface staining only. (TIF) [file pone.0102998.s001.tif]

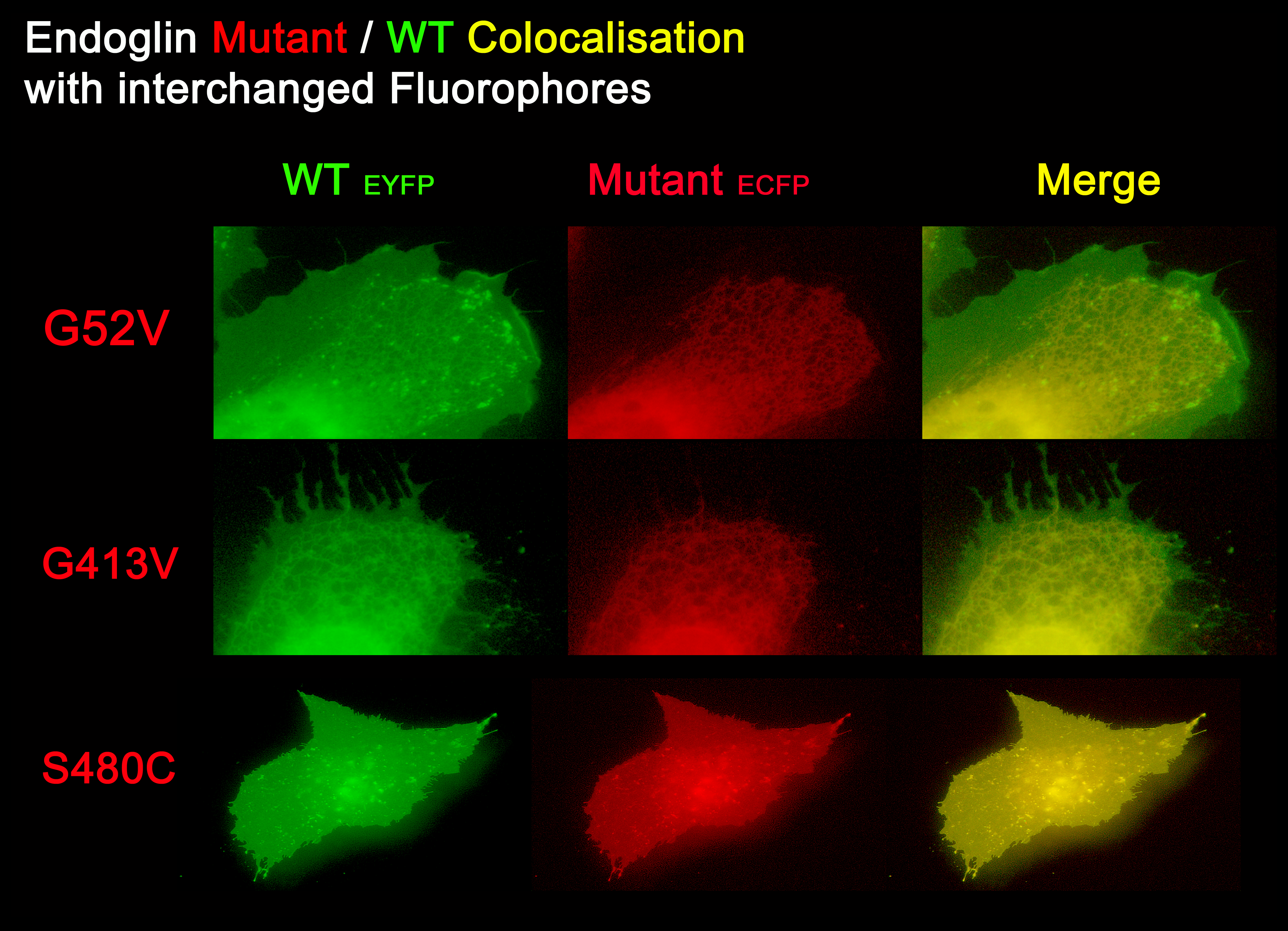

Supplement: Figure S2 — Colocalisation of endoglinwt and mutants visualized with interchanged fluorophores in CHO cells. CHO cells were co-transfected with endoglinwt-EYFP and ECFP-tagged mutants. This variation enhances visibility of the rER retained endoglinwt proportion through the plasma membrane but lowers visibility of the mutant proteins within the rER due to differences in fluorophore quantum yield (brightness). (TIF) [file pone.0102998.s002.tif]

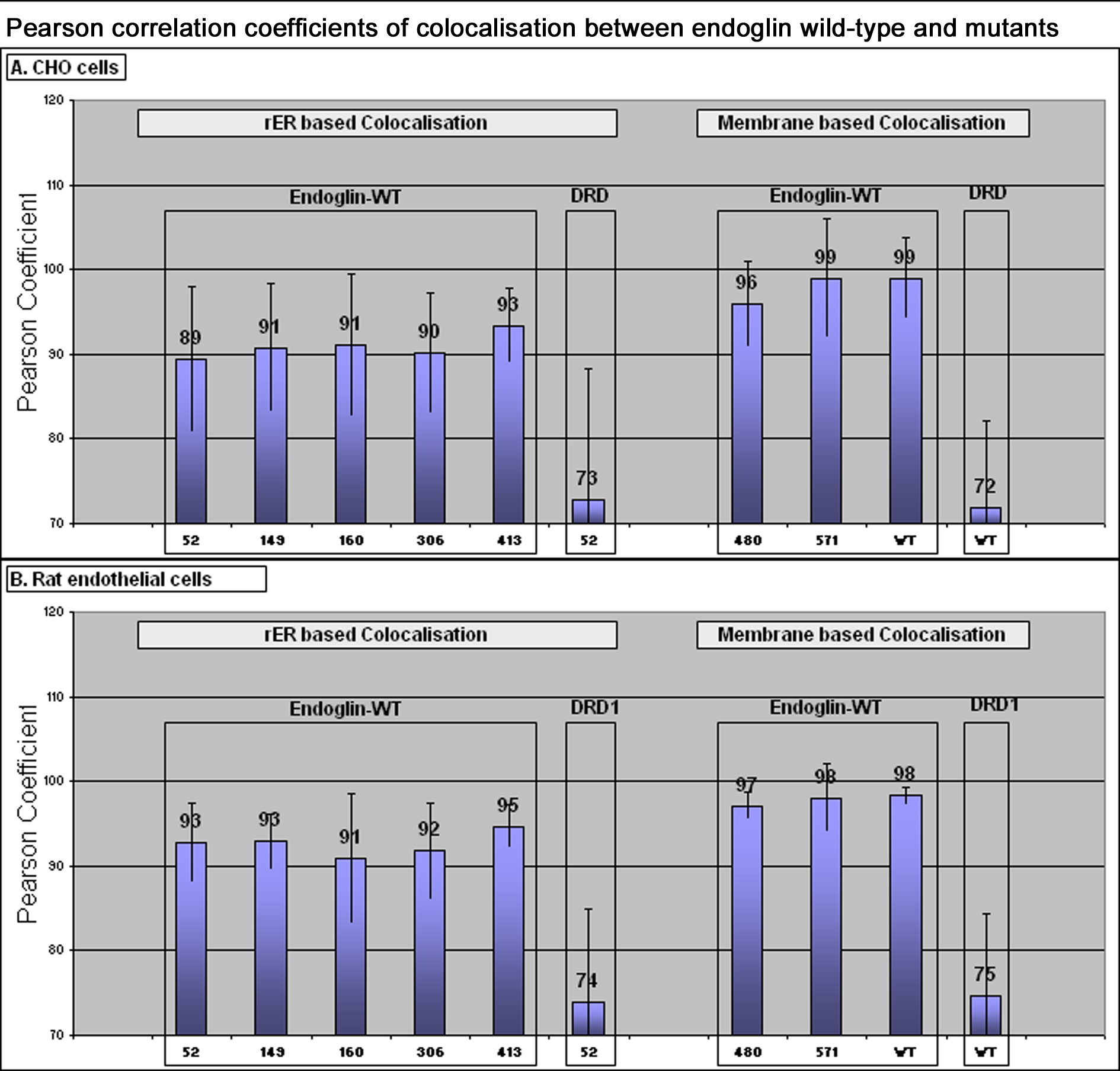

Supplement: Figure S3 — Quantitative colocalisation analysis of endoglinwt & mutants. Quantification of colocalisation between endoglin mutants and endoglinwt was performed using Pearson correlation coefficients of two channel fluorescence images. CHO cells were co-transfected as indicated and live cell images were taken after 24 hours of expression. Samples are shown in two different groups depending on localisation of the respective endoglin mutant proteins either in the rER or in the plasma membrane. Within the groups no significant differences can be observed among the mutants. However, membrane localised mutants produce stronger colocalisation values (∼99) with endoglinwt than ER localised mutant proteins (∼90). Co-transfection of the DRD1 receptor together with the ER trapped mutant G52V or together with endoglinwt results in a coefficient of ∼70. Av: Group average. The results represent mean values of three experiments. (TIF) [file pone.0102998.s003.tif]

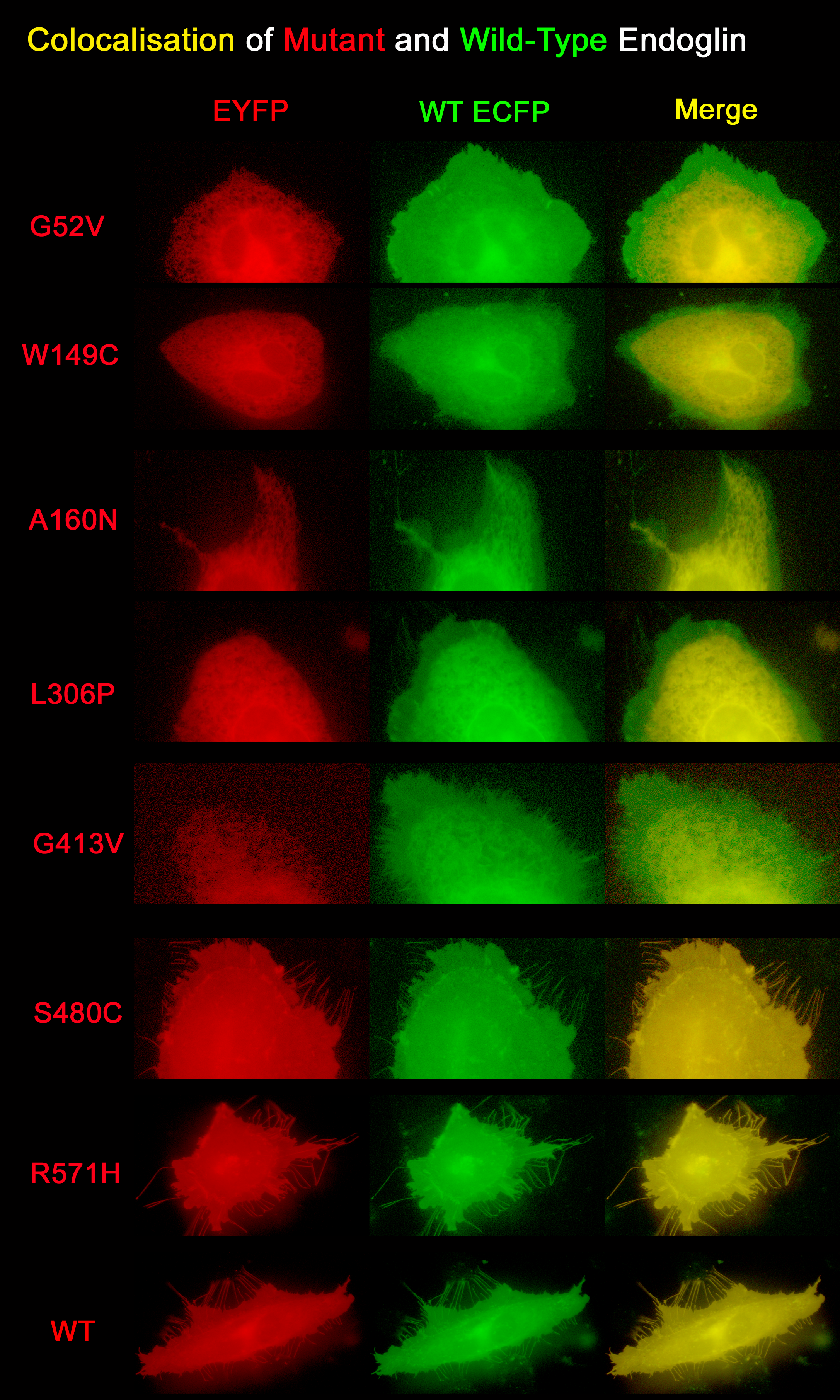

Supplement: Figure S4 — Colocalisation of endoglinwt and mutants in rat endothelial cells. Rat endothelial cells (RECs) were cotransfected with endoglinwt - ECFP and endoglin mutants (EYFP). The localisation of mutant proteins and endoglin wild-type in RECs is identical to the localisation as observed in CHO cells. (TIF) [file pone.0102998.s004.tif]

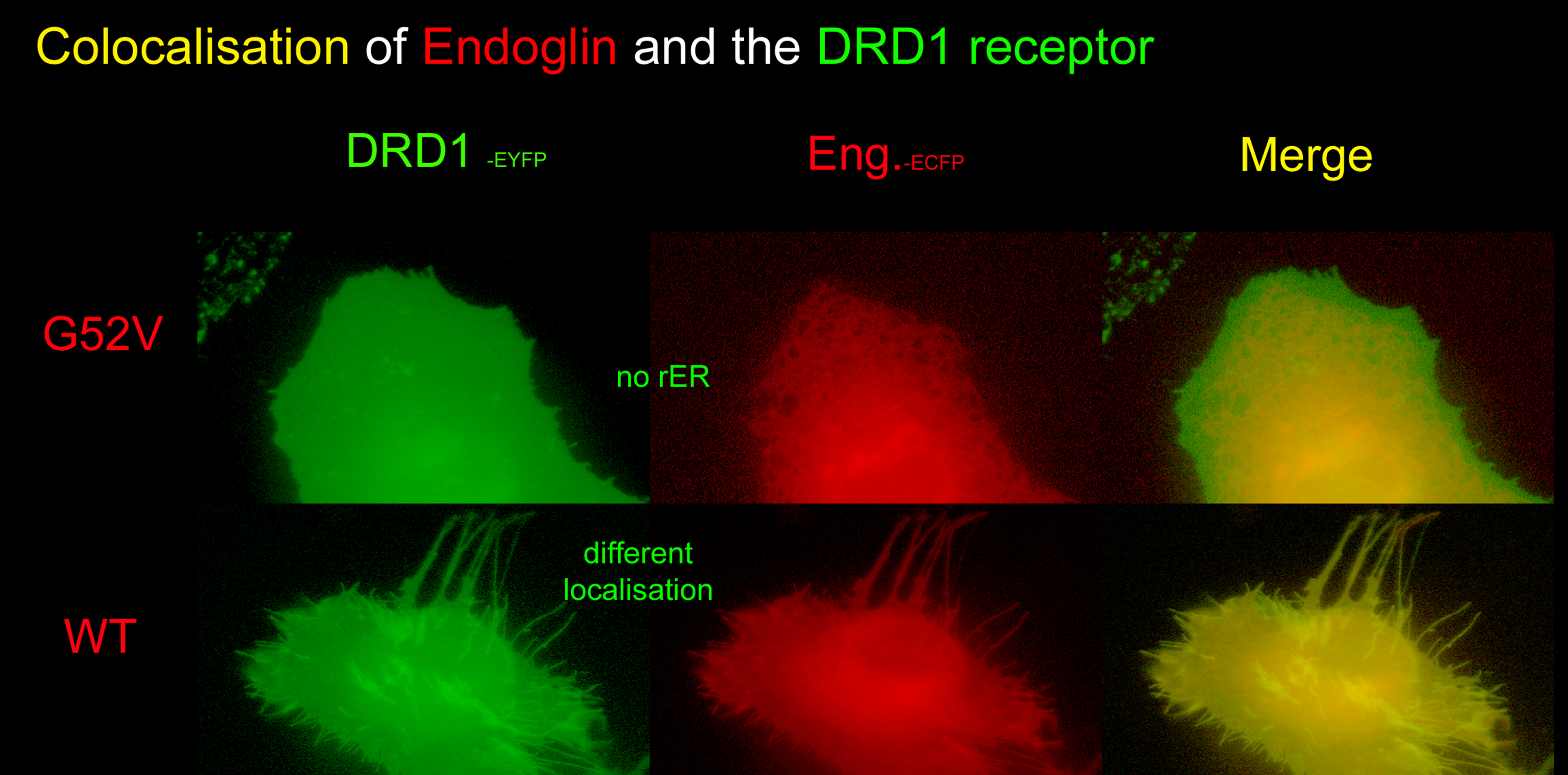

Supplement: Figure S5 — Co-transfection of endoglinwt and endoglinG52V with the dopamine receptor D1 (DRD1). CHO cells were co-transfected with endoglinwt-ECFP or endoglinG52V-ECFP together with the DRD1-EYFP expression construct. As displayed the DRD1 receptor is not retained in the rER by the G52V endoglin mutant protein. Furthermore DRD1 shows a different localisation pattern than the endoglinwt protein. This leads in both cases to lower Pearson correlation values as shown in figure S3. (TIF) [file pone.0102998.s005.tif]

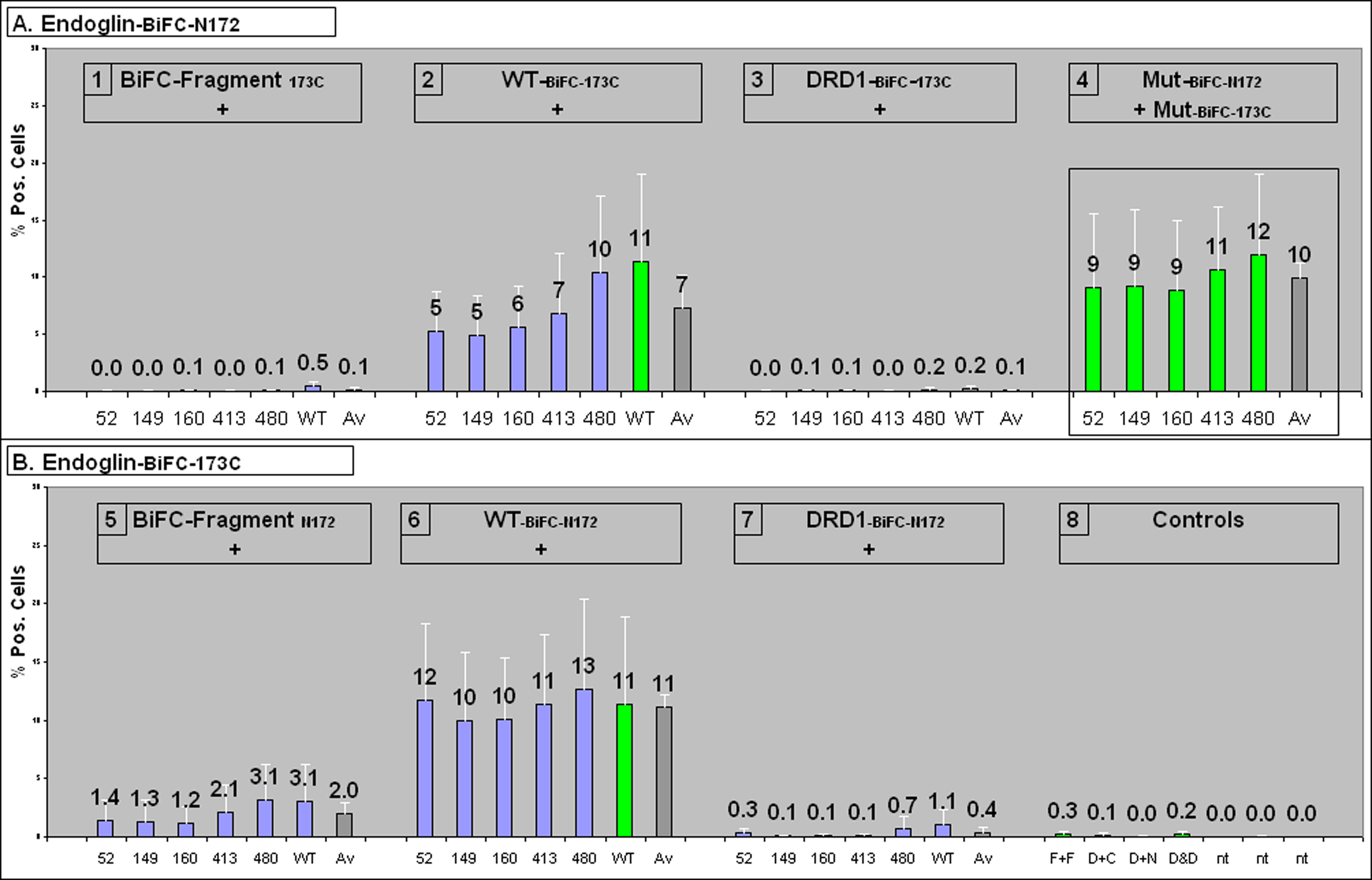

Supplement: Figure S6 — Determination of BiFC specificity through endoglin dimerisation by Flow-Cytometry. Technical details : In order to test the complementation specificity caused by endoglin dimerization against unspecific auto-complementation of the BiFC fragments, cells were co-transfected with the corresponding compatible BiFC partners as indicated and counted by flow cytometry after an expression time of 24 hours. The experiment was set up containing two different test variations. Variation A: The N-terminal BiFC fragment fused to the different endoglin variants was co-expressed with its C-terminal BiFC counterpart either alone [1] or fused to endoglinwt [2] or fused to the DRD1 receptor [3]. Variation B: The C-terminal BiFC fragment fused to the different endoglin variants was co-expressed with the corresponding N-terminal BiFC fragment as in variation A [5], [6], [7]. Endoglin homodimers (green, [2], [4], [6]) can not be classified within these two variations as interchanging BiFC fragments does not apply. Controls [8]: (F+F) putative auto-complementation by BiFC fragments alone. (D+C) DRD1 + C-terminal BiFC fragment. (D+N) DRD1 + N-terminal BiFC fragment. (D+D) putative auto-complementation of DRD1 receptors (DRD1 is a monomeric receptor). (nt) non-transfected cells as a gating control. The average (Av) for each group is indicated (grey bar). The diagramme shows mean values of 4 independent experiments. Result : Occurrence of BiFC produced by endoglin dimerisation is significantly higher than produced by intrinsic fragment auto-complementation [1], [5] or when expressed together with the DRD1 receptor [3], [7]. The auto-complementation background [5] becomes diminished (∼0) when co-expressing the DRD1 receptor BiFC counterpart [7], instead of co-expressing the unfused BiFC fragments [5], which resembles the artificial character using single BiFC fragments as a control for unspecific fragment auto-complementation. (TIF) [file pone.0102998.s006.tif]
